# Transcriptomic evidence that insulin signalling pathway regulates the ageing of subterranean termite castes

**DOI:** 10.1038/s41598-020-64890-9

**Published:** 2020-05-18

**Authors:** Haroon, Xiao-Ming Ma, Yu-Xin Li, Hong-Xin Zhang, Qing Liu, Xiao-Hong Su, Lian-Xi Xing

**Affiliations:** 1 Shaanxi Key Laboratory for Animal Conservation (Northwest University), Xi’an, 710069 China; 2 Key Laboratory of Resource Biology and Biotechnology in Western China (Northwest University), Ministry of Education, Xi’an, 710069 China; 3 College of Life Sciences, Northwest University, No. 229, North Taibai Rd., Xi’an, Shaanxi Province 710069 P.R. China

**Keywords:** Developmental biology, Ecology, Evolution, Molecular biology, Physiology, Zoology

## Abstract

Insulin is a protein hormone that controls the metabolism of sugar, fat and protein via signal transduction in cells, influencing growth and developmental processes such as reproduction and ageing. From nematodes to fruit flies, rodents and other animals, glucose signalling mechanisms are highly conserved. Reproductive termites (queens and kings) exhibit an extraordinarily long lifespan relative to non-reproductive individuals such as workers, despite being generated from the same genome, thus providing a unique model for the investigation of longevity. The key reason for this molecular mechanism, however, remains unclear. To clarify the molecular mechanism underlying this phenomenon, we sequenced the transcriptomes of the primary kings (PKs), primary queens (PQs), male (WMs) and female (WFs) workers of the lower subterranean termite *Reticulitermes chinensis*. We performed RNA sequencing and identified 33 insulin signalling pathway-related genes in *R. chinensis*. RT-qPCR analyses revealed that *EIF4E* and *RPS6* genes were highly expressed in WMs and WFs workers, while *mTOR* expression was lower in PKs and PQs than in WMs and WFs. PQs and PKs exhibited lower expression of *akt2-a* than female workers. As the highly conserved insulin signalling pathway can significantly prolong the healthspan and lifespan, so we infer that the insulin signalling pathway regulates ageing in the subterranean termite *R. chinensis*. Further studies are recommended to reveal the biological function of insulin signalling pathway-related genes in the survival of termites to provide new insights into biomolecular homeostasis maintenance and its relationship to remarkable longevity.

## Introduction

Animals have a limited life span from a few weeks (e.g., the fruit fly *Drosophila melanogaster*, the nematode *Caenorhabditis elegans*, and the water flea *Daphnia longispina*), up to living for centuries (e.g., the clam *Arctica islandica*)^1,2^. Lifespan extension has long been of interest, but the mechanisms contributing to longevity remain mostly unknown. Invertebrates such as fruit flies and nematodes and some vertebrates (fish, mice, etc.) with relatively short lifespans and gestation periods are excellent models for the study of longevity mechanisms. Interestingly, the development of technology, such as next-generation sequencing (NGS)^3^ has recently made novel animal models applicable to longevity studies.

The members of social insects living in a single colony, such as honey bees, ants and termites, exhibit variations in lifespan that can differ by two orders of magnitude^4,5^. Reproducing queens and kings of termites can live for 20 years, while non-reproducing workers only live for a few weeks to months. This phenomenon makes social insects particularly interesting new models of within-species variation in the rate of senescence. Additionally, all of the members of a termite colony have the same genetic background, and longevity variations are articulated by different gene expression, which may lead to exceptional longevity and a sustained reproductive capacity^6^. As the royal castes, queens and kings of termites are among the most promising subjects for ageing research, along with fruit flies and nematodes^7^.

Numerous studies on longevity and lifespan have been conducted using a variety of invertebrate and vertebrate species and have examined different metabolic pathways and molecules related to lifespan extension^8,9^. Growth factors such as growth factor-1, insulin and rapamycin have been extensively investigated, and sirtuin genes have been identified as genes that are upregulated in association with longevity in animals whose lifespan is extended due to caloric restriction^10^. Therefore, animals (e.g., *D. melanogaster*, *C. elegans*, *D. longispina* and *A. islandica*) that produce insulin-like peptides, mutations that reduce the levels or actions of these peptides may lead to significant increase in lifespan. The genetic control of aging and longevity of *C. elegans* results in more than 30 insulin-like peptides signalling the species via a common Daf2 receptor. It has been shown that the Daf2 gene exhibits significant homology to mammalian genes for the insulin receptor and the insulin-like growth factor 1 (IGF-1) receptor^11,12^. Consequently, insulin/IGF-1-like pathways also mediate metabolic effects relying on the winged-helix transcription factor, FOXO. In *C. elegans* FOXO is first identified as Daf-16, which elevates lipid level and longevity induced by Daf-2 loss^13^. Similarly, *D. melanogaster* has a homologous insulin/IGF-1-like pathway consists of insulin receptors, including mammalian insulin receptor substrates (IRS) and kinase homologs, and a forkhead transcription factor. These insulin receptors reduce signalling stress resistance and extend the longevity^14^. Senescence represents a decline fitness mechanism in age and life span, a highly plastic life-history trait that can be strongly influenced by the biotic and abiotic characteristics of the ambient environment^15^. As a result, the evolutionary and ecological importance of such declines in free-living populations and the intensively studied model laboratory systems is mainly unknown among individuals surviving into old age^16^. Molecular genetic studies have shown that insulin transduction regulates many physiological phenomena, such as the growth, development and longevity of insects^17^. The mutant insulin receptor substrate gene chico can significantly prolong the lifespan of female fruit flies^18^. In addition, the PI3K-Akt is the main pathway of the two-central insulin signalling pathways (ERK/MAPK and PI3K-Akt) in insects^19^, we, therefore, follow PI3K-Akt pathway to determine the insulin signalling pathway in the social insect of termite on the first time.

Previous studies of ageing in termites have focused mainly on antioxidant enzymes, DNA damage, and transposable element activity^20–22^. For example, the expression levels of DNA repair genes in the non-reproductive and primary reproductive states of *Reticulitermes speratus* were determined^19^, and several antioxidant enzyme genes were reported as potentially being associated with fertility and longevity in different castes^20^. Similar studies relating termite insulin signalling pathway-related genes and longevity have not yet been reported. The present study compared the expression levels of insulin signalling pathway-related genes among different castes of the subterranean termite *Reticulitermes chinensis*, including WMs, WFs, PQs and PKs, to reveal the mechanisms underlying different lifespans in these termite castes.

## Results

### Illumina data sequence and assembly

We established RNA-seq libraries using mRNA isolated from WMs, WFs, PQs and PKs of *R. chinensis*. A total of 109,126,456 clean sequencing readings were obtained via Hi-SeqTM 4000 (Illumina) paired-end sequencing. Each test provided more than 7 G of transcriptome data based on the clean reads, and the Trinity system assembled a total of 161,932 unigenes ranging from 201 bp to 19,428 bp (Supplementary Fig. 1). The mean length was 673 bp, with an N50 value of 933 bp. The performance evaluation based on GC showed an accurate approximation (42.90%). These findings suggest that the development and quality of the sequencing results were sufficient for further research.

### Functional annotation of the *R. chinensis* transcriptome

**Figure 1:**
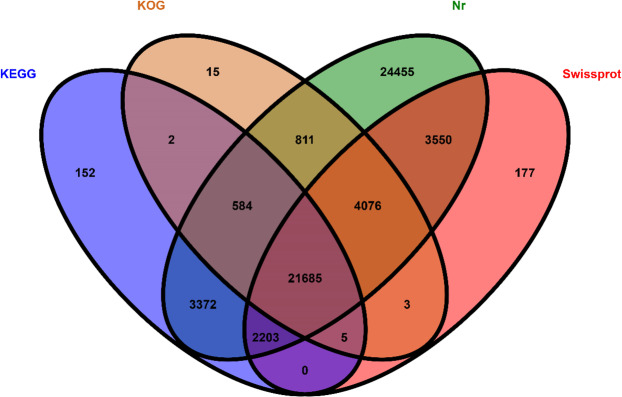
Venn diagram of the distribution of unigene and database matching results. The numbers of unique sequence-based annotations are the sum of the unique best BLASTX hits from the KEGG, KOG, Nr and Swiss-Prot databases. The overlapping regions between the four circles contain the numbers of unigenes that share BLASTX similarities in the respective databases.

**Figure 2:**
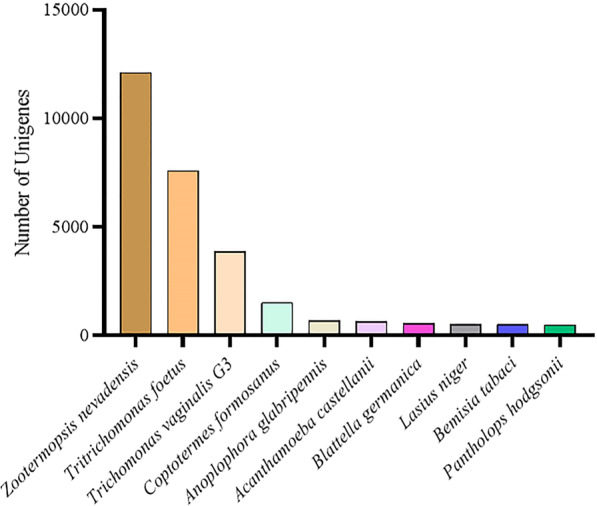
Species distribution of the BLASTX results. Using blastx to compare the assembled unigene sequences with the Nr database, the sequence with the best alignment (the lowest E value) of each unigene in the Nr database is the corresponding homologous sequence. The different colours represent different species.

For unigene annotation, we applied BLASTx (http://www.ncbi.nlm.nih.gov/BLAST/) with an e-value cut-off of 1e-5 to the NCBI nonredundant (Nr) protein database (http://www.ncbi.nlm.nih.gov), the Swiss-Prot protein database (http://www.expasy.ch/sprot), the Kyoto Encyclopedia of Genes and Genomes (KEGG) database (http://www.genome.jp/kegg)^23–25^, and the KOG database (ftp://ftp.ncbi.nih.gov/pub/COG/KOG/kyva). In total, 161,933 unigenes were annotated. The Venn diagram showed that the number of unique sequence-based annotations was the sum of the unique best BLASTX similarities from the Nr, Swiss-Prot, KOG and KEGG databases (Fig. 1). In the Nr database, 60,736 unigenes (37,51% of *R. chinensis* unigenes) presented significant matches, whereas in the Swiss-Prot database, 31,699 unigenes (19,58%), in the KOG database, 27,181 unigenes (16,79%), and in the KEGG database, 28,003 unigenes (17,30%) exhibited significant matches (Supplementary Table 1). Most of the sequences (100,843) were compiled, and 62.27% of the genes could not be identified due to the relatively short length of the distinct gene sequences and the lack of annotation of the *R. chinensis* genome information. These unigenes may also be *R. chinensis*-specific genes or short fragments that mainly come from untranslated regions (e.g., 5′ and 3′ UTRs) or nonconserved regions of protein-coding transcripts (Fig. 2)^26^. The experiment was designed for the whole termite, and the Nr full library was used for unigenes, where data on the intestinal protist could not be ruled out because it was impossible to determine whether the unigene annotated as a protist species was a termite or an intestinal protist gene, so it was kept during analysis.

### Gene Ontology (GO), Clusters of euKaryotic Orthologous Groups (KOG) and Kyoto Encyclopedia of Genes and Genomes (KEGG) ontology classifications

**Figure 3:**
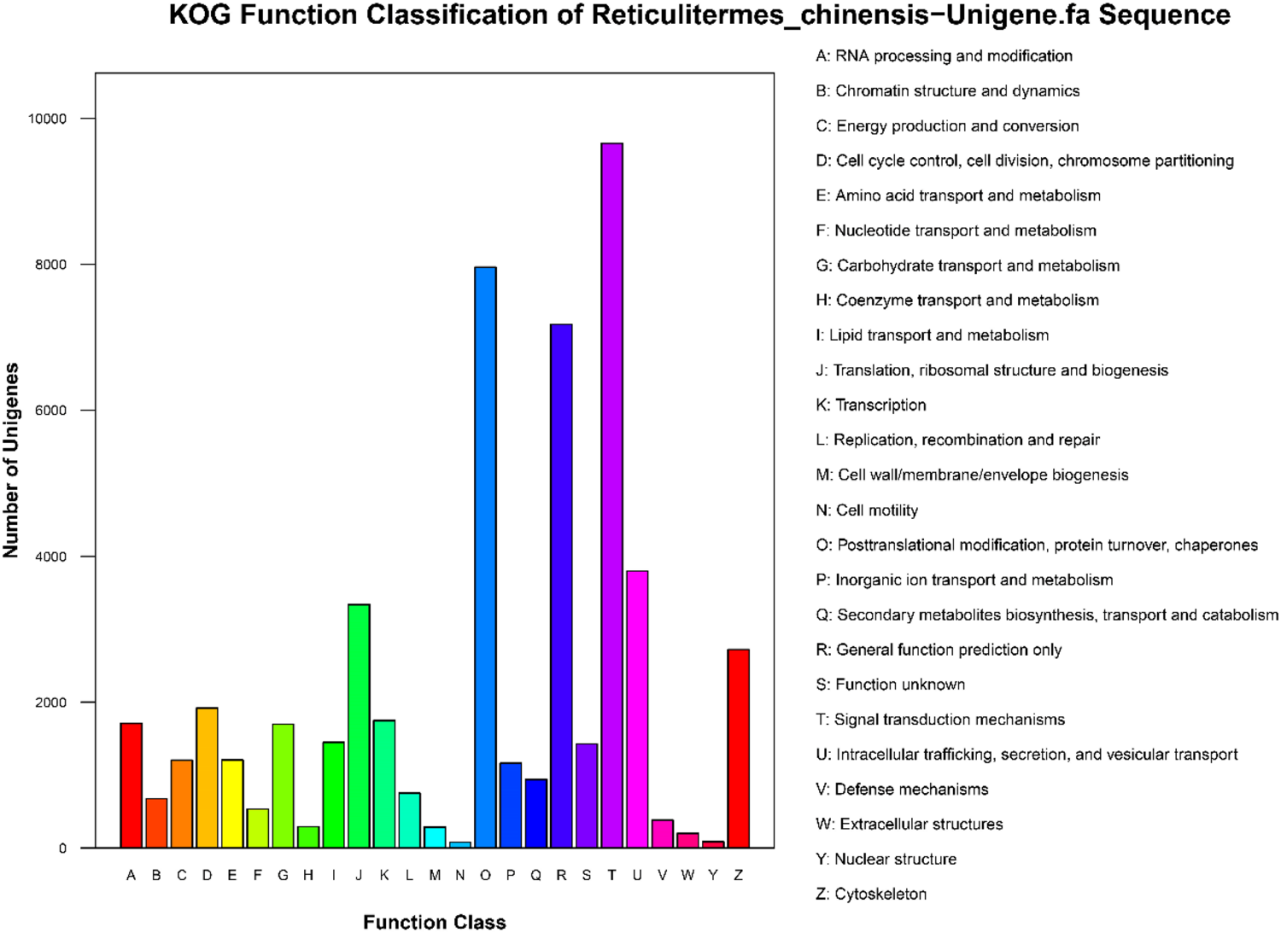
Histogram presentation of the Clusters of euKaryotic Orthologous Groups (KOG) classifications. A total of 52,430 unigenes were grouped into 25 KOG classifications. The y-axis indicates the number of unigenes in a specific functional cluster. The legend presents the 25 functional categories.

We classified the functions of the predicted unigenes using GO, KEGG, and KOG analyses based on the protein annotation results of Nr database homology searches. A total of 52,430 unigenes were annotated into 25 classes (Fig. 3) in the KOG functional classification.

**Figure 4:**
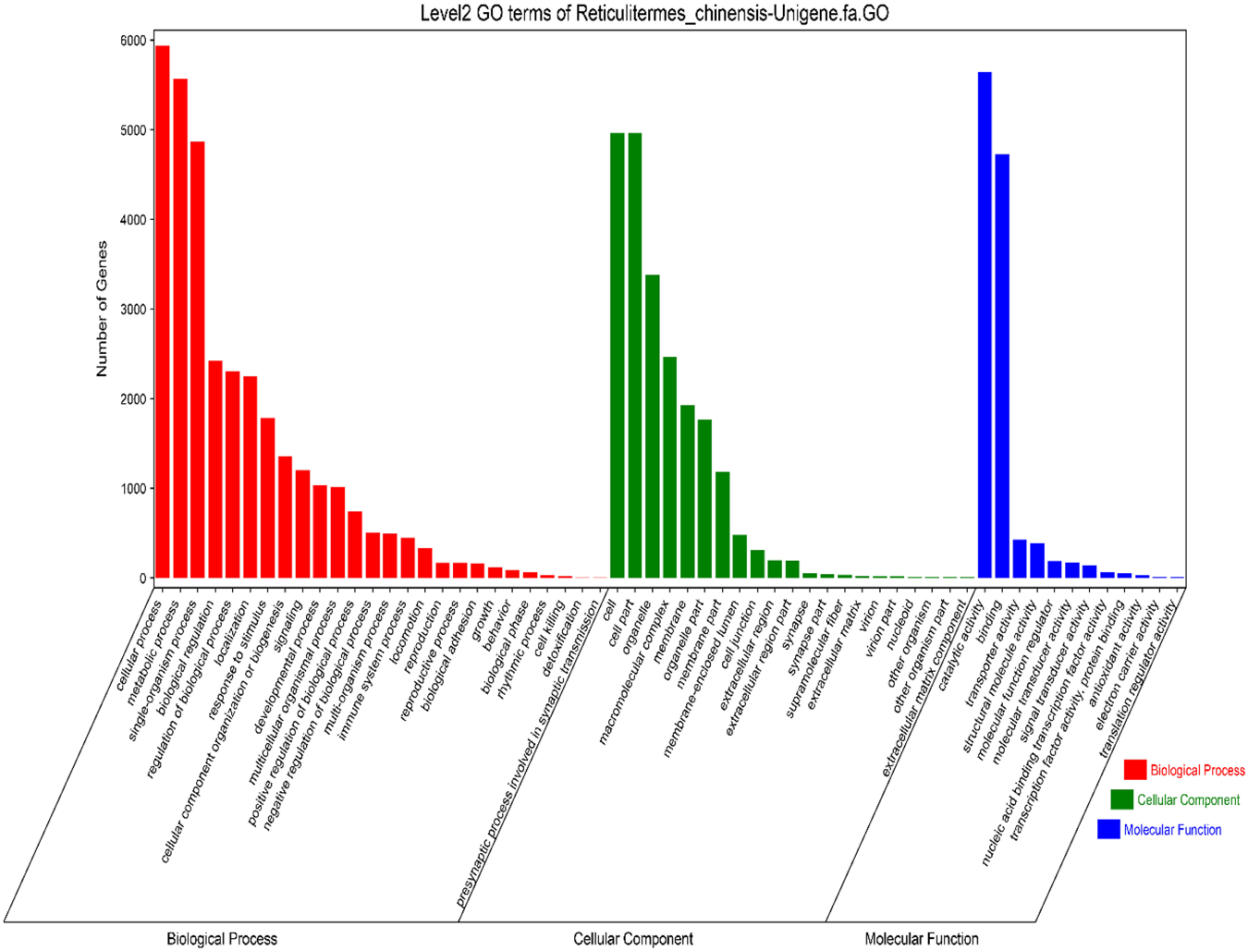
Histogram presentation of the Gene Ontology (GO) classification. The results are summarized in three main categories: biological process, cellular components, and molecular functions. The y-axis indicates the number of genes in a category.

**Figure 5:**
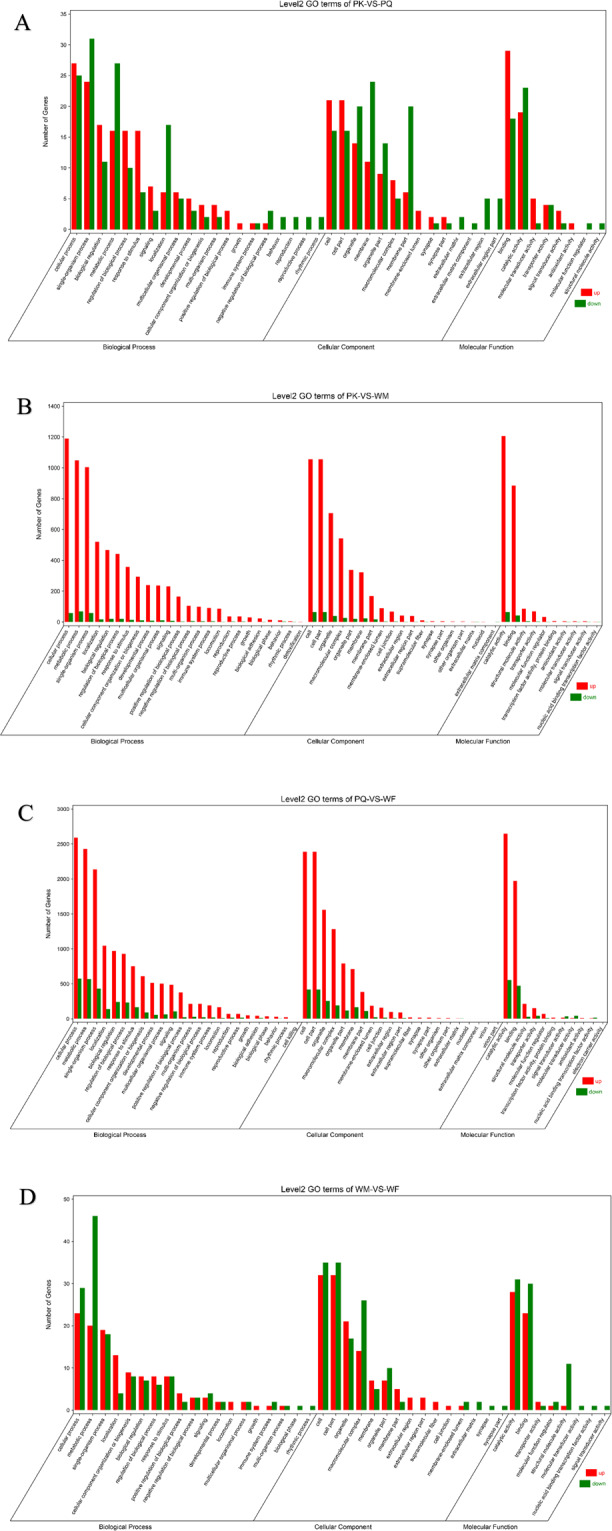
Histogram presentation of the Gene Ontology classification in each caste. (**A**), PK-VS-PQ; (**B**), PK-VS-WM; (**C**), PQ-VS-WF; (**D**), WM-VS-WF; PK, primary king; PQ, primary queen; WM, male worker; WF, female worker. The figure represents the up and down categorical presentation of biological processes, cellular components, and molecular functions. The x-axis indicates the names of genes in a category. The y-axis indicates the numbers of a specific category of genes in the main category.

To determine the biological functions of the DEGs among PKs, PQs, WMs and WFs, GO classification was carried out. A total of 59 categories (Fig. 4) of functional groups were analysed. Biological processes showed the highest probability density distribution of gene expression, followed by cellular components and molecular functions. All DEGs identified in this study were mapped to terms in the GO database to determine the functions of the differentially expressed genes. These included cellular processes, catalytic activity, metabolic processes, cell components, single-organism processes, and binding (Fig. 5A–D). Through sequencing, we found a total of 568 related genes involved in the insulin signalling pathway (Supplementary Fig. 4). There are 33 genes exhibiting significant differences in the primary kings, primary queens, and female and male workers of the lower subterranean termites. There are two insulin signalling pathways: the mitogen-active kinase (MAPK) pathway and the PI3K-Akt pathway. However, the PI3K pathway is the most prominent insulin signalling pathway in insects. Therefore, we selected 6 genes (*Pdk1, akt2-a, Tsc2, mTOR, EIF4E*, and *RPS6*) in the PI3K-Akt pathway showing significant differences.

**Figure 6:**
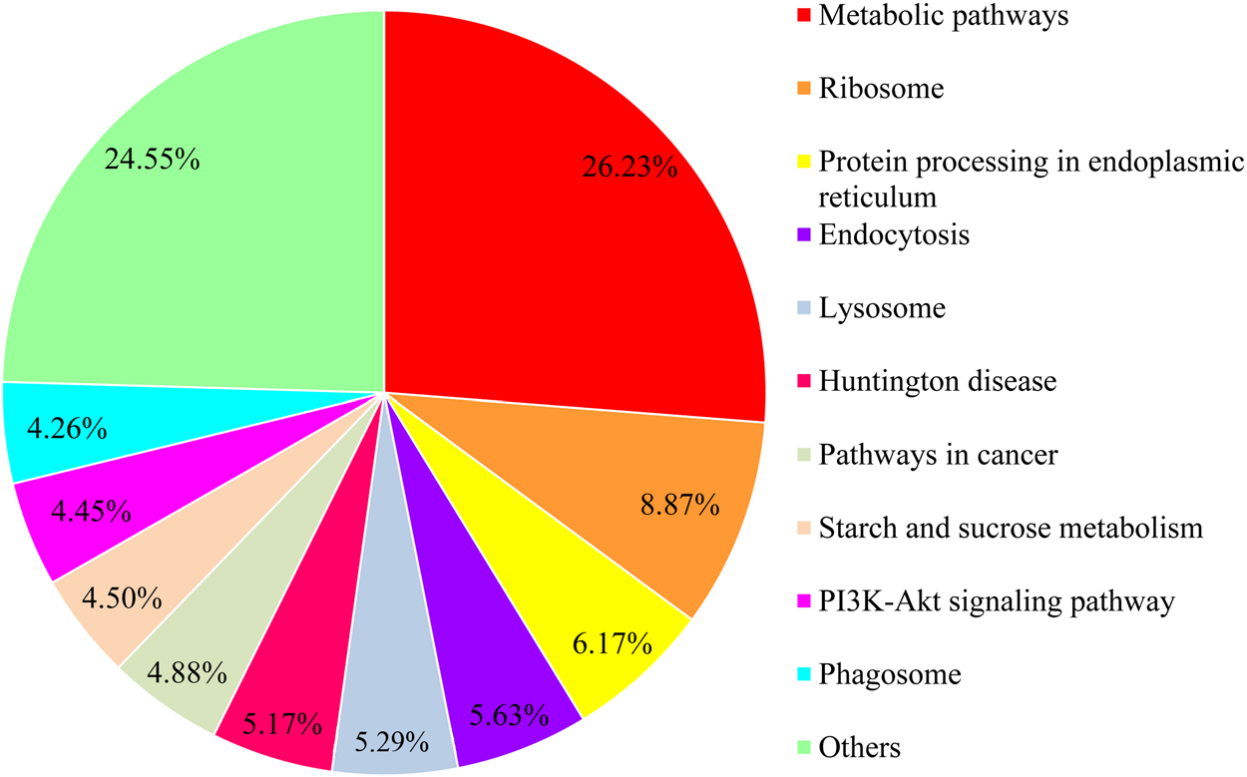
KEGG classifications of the unigenes. 19,291 unigenes were assigned to 343 pathways. The figure shows more than 800 unigenes annotated pathways.

To understand the biological pathways that are involved in *R. chinensis*, we mapped the unigene sequences to the reference canonical pathways in the KEGG database. A total of 19,291 sequences were assigned to 343 KEGG pathways. They were related to metabolic pathways (26.23%), ribosomes (8.87%), protein processing in the endoplasmic reticulum (6.17%), endocytosis (5.63%), lysosomes (5.29%), Huntington disease (5.17%), pathways in cancer (4.88%), starch and sucrose metabolism (4.50%), the PI3K-Akt signalling pathway (4.45%), phagosomes (4.26%) and other pathways (24.5%) (Fig. 6).

### Differentially expressed gene (DEG) analysis in WFs, WMs, PQs and PKs

**Figure 7:**
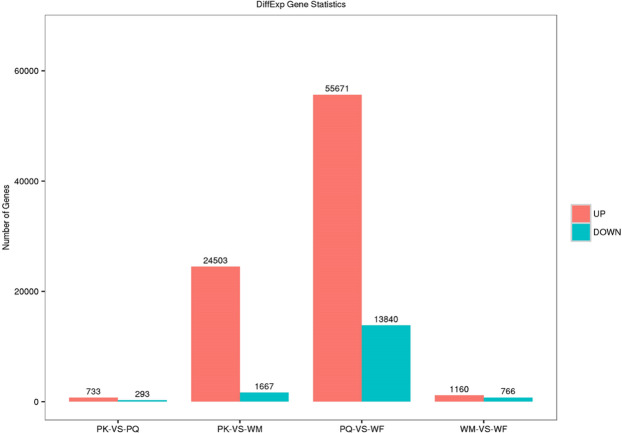
DEGs enrichment trend analysis across different castes. PK, primary king; PQ, primary queen; WM, male worker; WF, female worker. The column indicates the DEGs enrichment expressed genes in the PK, PQ, WM and WF, where the red column represents significantly up-regulated and the green column indicates significantly down-regulated. The parameters FDR ≤ 0.001 and log2Ratio ≥ 1 were used as the thresholds to judge the significance of gene expression differences.

**Figure 8:**
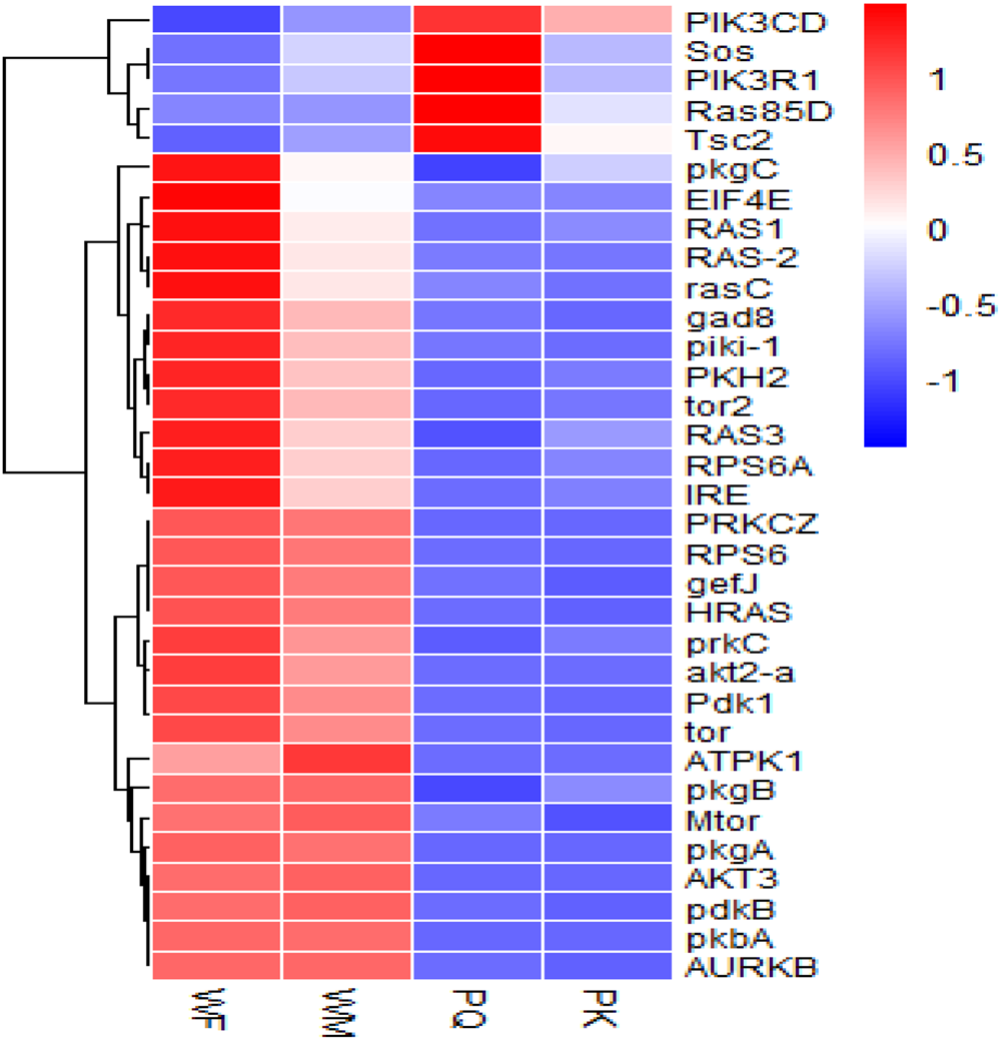
Heat map of the DEGs for the 33 genes involved in the insulin signalling pathway in different castes. PQ, primary queen; PK, primary king; WF, female worker; WM, male worker.

The DEGs (differentially expressed genes) were calculated (upregulated and downregulated genes) via the reads/fragments per kilobase of transcript per million mapped reads (RPKM/FPKM) method. A total of 98,633 DEGs were analyzed from PKs, PQs, WMs and WFs. The number of upregulated DEGs was 82,067 (83.20%), and that of downregulated DEGs was 16,566 (16.80%) (Fig. 7). The greatest number of upregulated DEGs were identified in PQ-VS-WF, at 55,671 (67.84%), followed by PK-VS-WM, at 24,503 (29.86%). The greatest number of downregulated DEGs were identified in PQ-VS-WF, at 13,840 (83.54%), followed by PK-VS-WM, at 1,667 (10.06%). The sequence analysis and annotation provided valuable information for the evaluation of all unigenes in the transcriptomes of *R. chinensis* WMs, WFs, PQs and PKs. The upregulated and downregulated genes between the WMs, WFs, PQs and PKs were used as filtering thresholds and for the identification of genes. The express transcriptome review showed that the expression of 33 genes was linked to the insulin signalling pathway in WMs, WFs, PQs and PKs (FDR ≤ 0.05; Fig. 8). A comparative analysis of downregulated genes revealed a total of 31 DEGs genes in PKs.

### Protein-coding region prediction (CDS)

The protein-coding regions (CDSs) of all unigenes were predicted to explore the functions of the unigenes at the protein level. In addition, BLASTX (value < 0.00001) and ESTScan (version 3.0.2) were used to estimate 61,075 and 37,514 unigenes, respectively. The length and distribution of the CDSs predicted in the BLAST and ESTScan tests, respectively, are shown in histograms in Supplementary Figs. 2 and 3. Generally, when the length of the series increased, the number of CDSs decreased gradually. This finding is consistent with the outcome of the unigene assembly.

### Caste-specific expression analysis of insulin signalling pathway-related genes

**Figure 9:**
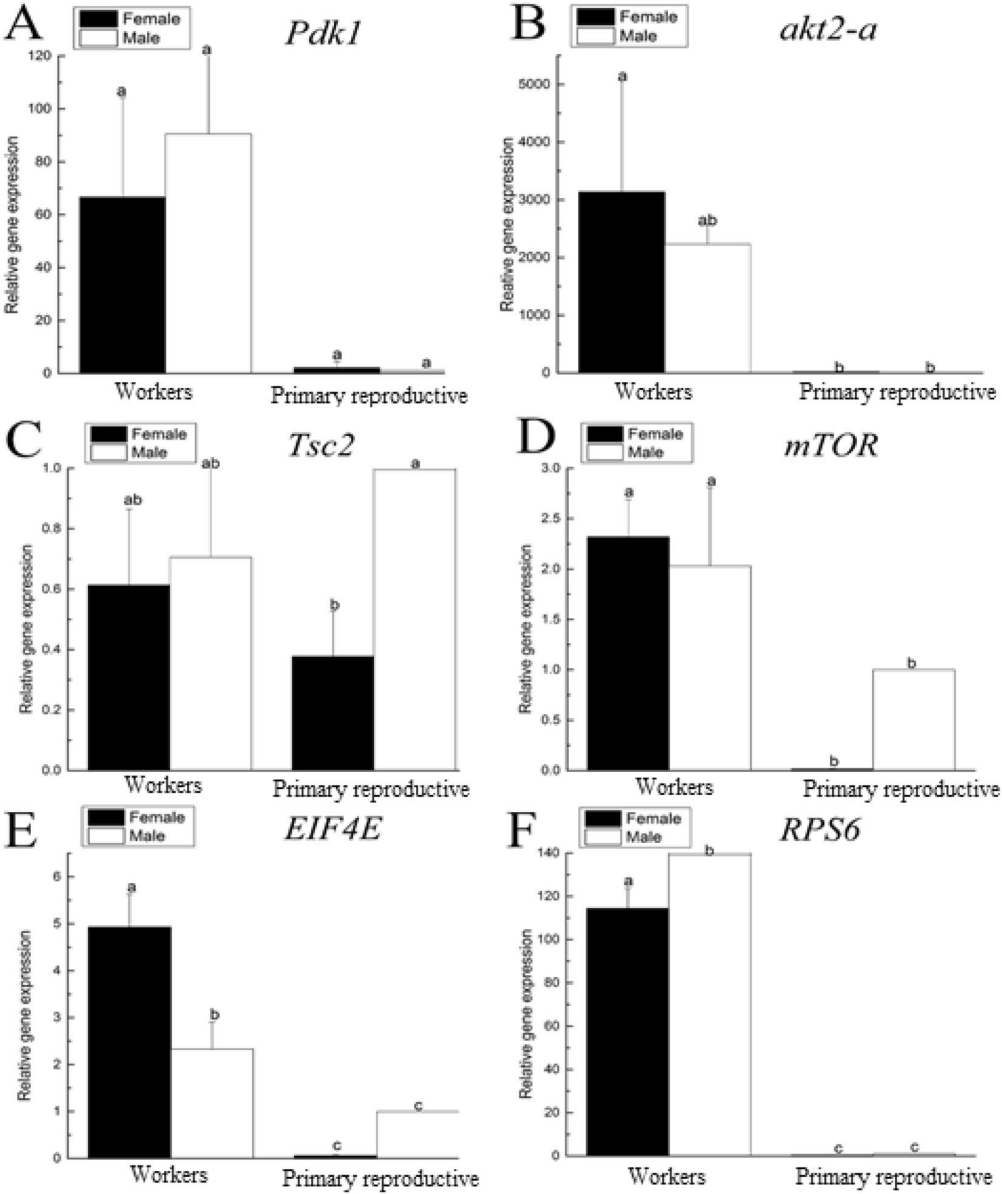
The expression of insulin signalling pathway-related genes in workers and primary reproductives. (**A**), *PdK1*; (**B**), *akt2-a*; (**C**), *Tsc2*; (**D**), *mTOR*; (**E**), *EIF4E*; (**F**), *RPS6*. The x-axis indicates the four different castes. Black and white bars indicate female and male individuals, respectively. Error bars represent the standard error of the mean. Different letters (a–c) over the bars denote significant differences at P < 0.05 (Mean ± SE, n = 5).

The RT-qPCR analysis was performed to investigate insulin signalling pathway-related gene expression among WMs, WFs, PQs and PKs. We focused on the phosphoinositide-dependent kinase-1 (*Pdk1*), protein kinase B2 (*akt2-a*), tuberous sclerosis-2 (*Tsc2*), mammalian target of rapamycin (*mTOR*), eukaryotic translation initiation factor 4E (*EIF4E*) and ribosomal protein S6 (*RPS6*) genes. These genes play significant roles in the insulin signalling pathway among different castes of termites. The primers for each unigene are provided in Supplementary Table 2. We evaluated the expression levels of *Pdk1*, which were somewhat lower in PKs and PQs, but there were no statistically significant differences (Fig. 9A–F) among different castes (n = 3, P= 0.04< 0.05; Fig. 9A). The RT-qPCR results revealed that PQs and PKs presented lower expression of *akt2-a* than WFs (n = 3, P = 0.025 < 0.05; Fig. 9B). In addition, *Tsc2* expression was higher in reproductive than non-reproductive castes (n = 3, P = 0.05 ≤ 0.05; Fig. 9C). In contrast, *mTOR* expression was lower in PKs and PQs than in the WMs and WFs castes (n = 3, P = 0.019 < 0.05; Fig. 9D). Furthermore, the expression levels of *EIF4E* (n = 3, P = 0.015 < 0.05; Fig. 9E) and *RPS6* (n = 3, P = 0.016 < 0.05; Fig. 9F) were significantly lower in PQs and PKs than in WMs and WFs.

**Figure 10:**
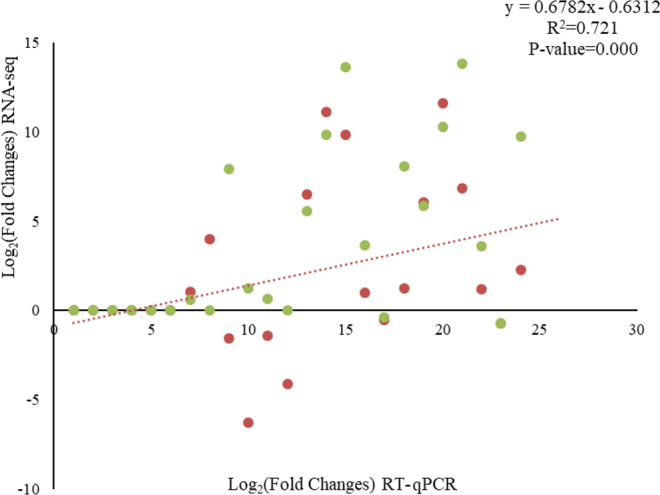
The correlation of the expression level of six insulin signalling pathway-related genes between RNA-Seq and RT-qPCR in workers and reproductives.

The RPKM and RT-qPCR validation was performed for 6 genes found to be differentially expressed in the scatter plot correlation among the WMs, WFs, PQs and PKs castes. The correlation coefficient between the RT-qPCRs and transcriptome data validation results was 0.721 (Fig. 10).

## Discussion

Age verification or determination is a requirement for the identification of individuals in living organisms and for the prediction of death. *Drosophila melanogaster* and *Caenorhabditis elegans* are well-established research organisms in the field of ageing research. These organisms may not only exhibit a prolonged life but may also be healthier throughout their life span. Studies among social insects, including termites, show that there is diverse caste differentiation (workers, soldiers, king and queen) within a single colony. The queen and king can live for 20-30 years, while non-reproductive castes only live for a few weeks to months. The specific associated developmental, biological and physiological changes in a given cell or tissue type have been examined through the evaluation of transcriptome sequences^19,27^. Transcriptomics refers to the collection of all RNA molecules, from protein-coding (mRNA) to non-coding RNAs, including rRNAs, tRNAs, lncRNAs, and pri-miRNAs among others, for which specialized library preparation methods and appropriate bioinformatic data processing and abundance quantification techniques are required for functional analysis^26^.

To understand global changes in the expression of thousands of genes, transcriptome analysis requires an effective statistical method with multiple comparison tests. The accuracy of quantitative RT-qPCR and microarray analyses has become evident in recent years and is highly dependent on the selection of genes for standardization. However, the present study is the first on termite insulin signalling pathway-related genes. From the transcriptome data, we obtained a total of 33 DEGs involved in the insulin signalling pathway by producing a high-quality dataset from *R. chinensis* (Fig. 6). These genes (*Pdk1, akt2-a, Tsc2*, *mTOR, EIF4E*, and *RPS6*) are involved in the insulin signalling pathway in different insects (i.e., *Drosophila*)^28^. Rintelen^29^ investigated whether *in vivo Pdk1* presents more than one target and functions in *PI(3)K* signalling at the downstream level in *C. elegans*. In addition, many *in vitro* invertebrate cell culture studies have indicated that *Pdk1*, in the AGC family of kinases (consisting of *Akt, S6K, RSK, PKN* and all protein kinase C isoforms), is the critical regulator of T-loop phosphorylation. *Pdk1* has two active domains, an amino-terminally bound serine-threonine kinase domain and a pleckstrin homology domain with excessive PIP3 affinity. *Pdk1* is the direct effector of *Akt, S6K*, and *RSK*, and the activation of all three of these kinases is blocked in *Pdk1*-deficient embryonic stem cells. However, previous genetic evaluations of *C. elegans* and *Drosophila* were primarily based on the finding that *Akt* plays a significant role in *Pdk1* activity as a central regulator of cell growth through AGC kinases *Akt* and *S6K*, respectively^28,29^. Recently, Gao *et al*.^30^ examined the role of *PI3K-Akt* and FOXO proteins in insulin signalling pathways in *Sogatella furcifera*. They reported physiological and genetic changes in the wing patterning of *S. furcifera*.

A similar study was reported in *C. elegans* indicating that *Pdk1* regulates the physiological effects of insulin and growth factors by activating a series of kinases controlling cell growth, differentiation, survival, protein translation and glucose metabolism. If inactivation of *Pdk1* occurs, *C. elegans* will enter the stagnant dauer stage and extend its lifespan^31^. This phenomenon (inactivation of *Pdk1*) was also reported in primary reproductives (king and queen). The results of the present study regarding the expression levels of *Pdk1* in different termite castes showed that there was no statistically significant difference between reproductive and worker termites. Consequently, our results indicated that the function of *Pdk1* in regulating termite lifespan showed the same changes as in nematode and other insects^28,30^. Therefore, the low expression^31^ (*C. elegans*) of *Pdk1* had little effect on the longevity or physical morphology of the termites. The *Pdk1* induced bent wing phenotype in *S. furcifera* depends on average levels of *dS6K* and *Akt*, because null mutations in either of the corresponding genes dominantly suppress the longevity. These findings, together with biochemical evidence from cultured cells showing that *Pdk1* regulates the activity of *Akt* and *dS6K*, provide functional evidence that *Pdk1* is a key regulator of growth and cell size by controlling the activity of two AGC kinases, *Akt* and *dS6K*^29^. The a*kt2-a* belongs to a subfamily of serine/threonine protein kinases consisting of three members, a*kt1*, a*kt2* and a*kt3*. The activation of these genes involves a combination of numerous stimuli and the activation of hormones, metabolism, growth factors and cell motility^32,33^. In the classical control model of *Akt1*, the PIP3 phospholipid recruits *akt1* to the plasma membrane, where two protein kinases, mTOR and Pdk1, phosphorylate Akt1 at its C-terminus and activation loop, respectively. The dual phosphorylation of *Akt1* results in increased kinase activity targeting protein substrates such as GSK3 and FOXO. The activity of *Akt1* may promote cell growth, block apoptosis, and mediate the insulin response, and clinically produced *akt* inhibitors include binding compounds targeting the ATP site and allosteric site^34,35^. Our findings suggest that the low expression of *akt2-a* in the primary reproductive castes may also prevent tumour cell invasion and metastasis. A related cell mutation study was conducted for the *Tsc1 Drosophila* homologue via mosaic screens, and the size of *Tsc1* mutant cells was significantly increased. The body volume was also increased, specifically by the tissues that contained the most mutant cells. Mutations in the *Drosophila*, *Tsc2* gene have previously been shown to induce similar phenotypes, and it has been proposed that polyploidy triggers the switch in the cell. Clones of *Tsc1* mutant cells undergo certain divisions within imaginal disks; however, they retain normal ploidy. The ectopic overexpression of *Tsc1* or *Tsc2* in *Drosophila* tissue does not inhibit but competes with the expression of *Tsc1* and *Tsc2*, leading to a great decline in cell growth and proliferation^36^. *Tsc1* and *Tsc2* are genes responsible for the suppression of tumours that contain a protein called tuberin^37^. *Tsc2* is widely distributed across cell types and organ systems, and these genes and proteins are highly conserved in interspecies sequences from *Drosophila* to humans. *Tsc2* binds to a third protein, TBC1D7, as part of a heteromeric protein complex to control cell growth, cell size, the cell cycle, and mTOR pathway proliferation. In contrast, large deletions, indels, nonsense and missense mutations and splicing errors are included in *Tsc2* mutations^38^. The hypothesized findings (RT-qPCR results) were obtained in the present study from the reproductive castes, in which higher expression of *Tsc2* was identified than in the non-reproductive castes of this termite.

Ageing is described as an accumulation of cellular damage over time promoting disease and death^39^. The germline genome is vulnerable to the collection of deleterious mutations throughout meiotic DNA replication. If a mutation is not eliminated from reproductive cells, it can be passed on to offspring, which is linked to an elevated threat of diseases in future generations. When mutations occur in somatic cells, they cannot be transmitted to the next generation. However, if mutations occur in the gametes of an organism, they may affect the offspring^40^. The mammalian target of rapamycin (*mTOR*) has been reported in many organisms, such as yeast and mammals. *mTOR* belongs to the serine/threonine kinase family of phosphatidylinositol kinase-related kinases (PIKKs)^41^ and contributes to increases in growth factors, physiological processes, cell metabolism and survival, and autophagy. The basal amount of *mTOR* leads to a low fecundity rate, egg size, and follicle numbers and is correlated with low vestigial (Vg) expression in *Aedes aegypti*, *Nilaparvata lugens*, *Drosophila* and *Apis mellifera*^42,43^. Accumulating evidence suggests that aberrant regulation of both cell growth and metabolism substantially contributes to cancer improvement and progression^44^. Therefore, earlier research has indicated that the high expression of *akt2-a, mTOR, EIF4E*, and *RPS6* is closely associated with cancer, growth factors, reproduction, physiological processes (wing patterning of *S. furcifera*)^30^, the inflammatory response, cell survival, weight problems and autophagy^45–48^. Furthermore, previous researchers discovered that the high expression of the breast cancer susceptibility gene *BRCA1* leads to a long life in insects^49,50^. Hence, an efficient antioxidant system can prevent the accumulation of detrimental DNA changes and contribute to the longevity of termite kings. Long-lived reproductive individuals exhibit a stable defence system against transposons as a possible source of DNA damage, in contrast to short-lived workers of the termite *Macrotermes bellicosus*^51^. The present experimental results indicated that the expression of cancer-related genes is low in the primary reproductive castes of termites. They are unlikely to experience DNA damage because cancer incidence and longevity are associated with multi-gene regulation processes^52–59^. Consistent with these studies, our results suggest that extreme evolutionary pressures (body changes over time)^4^ potentially led to the low expression of insulin signalling pathway-related genes in primary reproductive castes of *R. chinensis*.

In *D. melanogaster* and *C. elegans*, the most prominent role of the insulin signalling pathway is to control longevity, puberty, growth and body size. The ability to generate observable aberrant phenotypes through the perturbation of cell growth has allowed rapid progress in growth regulation studies. These species show strong similarities in their food reactions and sensory compensation associated with insulin signalling pathways and insulin-like peptides. The insulin signalling pathway also controls the metabolic pathway in the fly. Interestingly, flies exhibit female sterility, increased triglyceride levels and an extended diapause life stage as physiological responses to harsh environmental conditions such as a low nutrient supply or low temperature. Increased stress tolerance often occurs during diapause, which together with greater energy reserves, increases longevity and therefore the probability of reproduction^60–62^. Similarly, the results provide a valuable resource for the study of ageing mechanisms, structures and related pathways. These findings suggest that relatively conserved proteins alter the insulin signalling pathway and considerably prolong the lifespan or even prevent the development of diseases related to ageing. Further studies are needed to reveal the biological function of insulin signalling pathway-related genes in the survival of termites using genetic tools such as RNA interference and transgenic constructs^63–65^. Ultimately, our results provide new insights into biomolecular homeostasis maintenance and its relationship to remarkable longevity.

## Materials and Methods

### Sample collection

**Figure 11:**
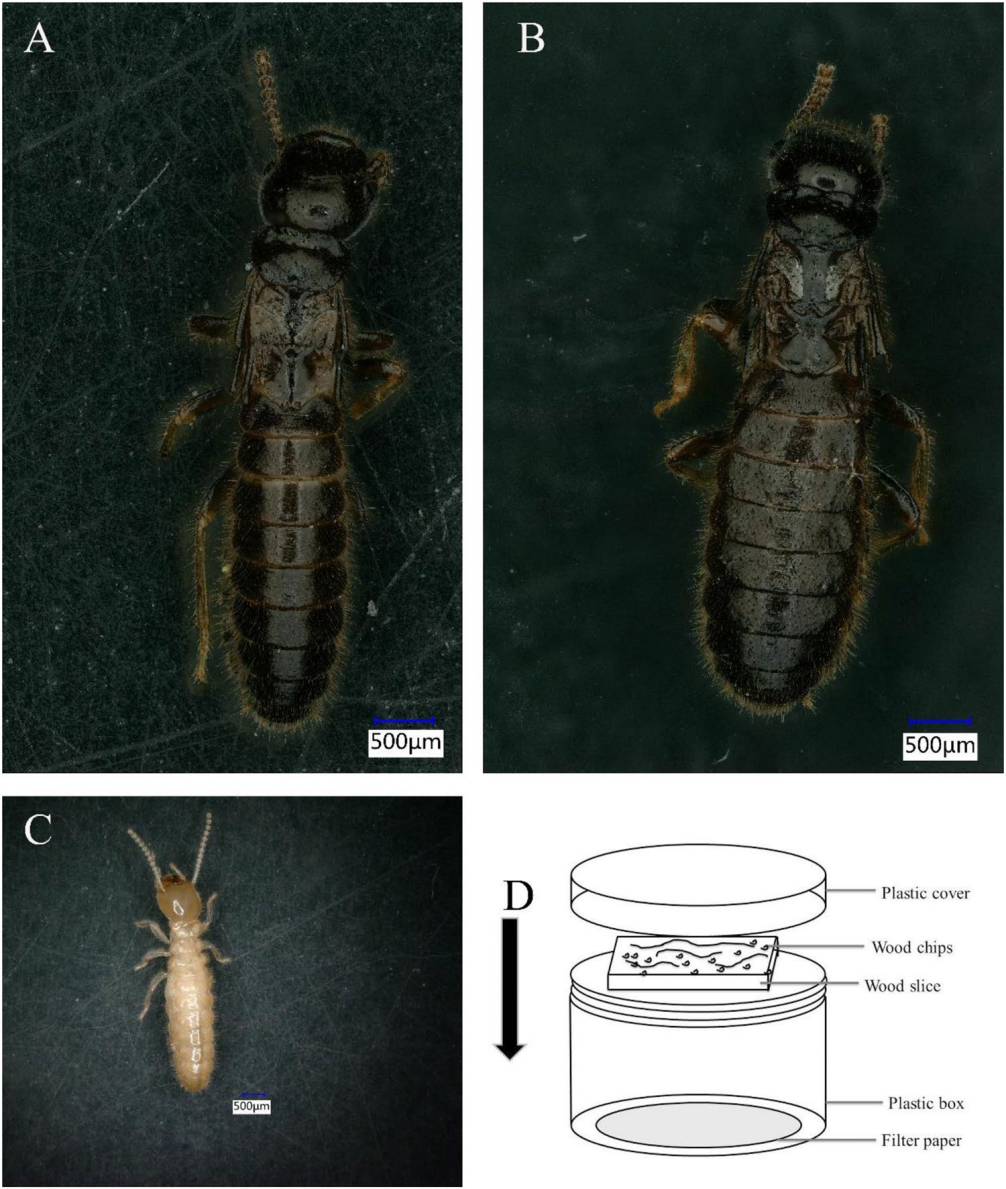
The morphology of the different castes of the *Reticulitermes chinensis* and rearing apparatus. (**A**), primary king; (**B**), primary queen; (**C**), worker; (**D**), rearing apparatus.

Alates of *R. chinensis* were collected from colonies in Chengdu in April 2014. The initial founder colony (each colony consisted of one male and one female alate) was by rearing a randomly selected male and female alate in a plastic box (80× 65× 40 mm) with damp chips of sliced pine wood at 25 °C in the laboratory (Termite House of Northwest University, China) (Fig. 11D). Individuals were extracted from each early-stage colony after 4 years, and they were regarded as PKs, PQs, and WMs and WFs workers (Fig. 11). As biological replicates for the experimental procedure, the whole bodies of the termites were temporarily preserved at −80 °C in liquid nitrogen. The WMs, WFs, PQs and PKs were separated (as a male and female) based on the characterization of the seventh sterna under an anatomical microscope. *Reticulitermes chinensis* is not an endangered or protected species; thus, no specific permits were required for the sampling and experiments.

### RNA extraction and Illumina sequencing

To obtain enough RNA, from PQs, PKs, WMs and WFs samples using TRIzol reagent and checked the total RNA quality assessed an Agilent 2100 Bioanalyzer (Agilent Technologies, Palo Alto, CA, USA). Three replicates of each caste WMs, WFs, PKs and PQs, were pooled to obtain enough RNA and build cDNA libraries using the NEBNext Ultra RNA Library Prep Kit for Illumina (NEB). In brief, using Oligo(dT) beads to extract the total RNA, enriched from mRNA and fragmentation buffer were used to transcribed into short fragments into cDNA with random primers. Second-strand cDNA was synthesized by using DNA polymerase I, RNase H, dNTPs and buffer. The cDNA fragments were then purified with the QiaQuick PCR extraction kit, poly(A) end-repaired was tailed, and attached to the Illumina sequencing adapters. Ligation products were selected in size by agarose gel electrophoresis, PCR amplified and sequenced using the Illumina HiSeqTM 4000 platform by Gene Denovo Biotechnology Co. (Guangzhou, China)^66,67^.

### *De novo* transcriptome assembly

The remaining reads from all samples were assembled using the Trinity version of trinityrnaseq r2012–04–27^68,69^, which generates transcriptomic assemblies from short-read sequences using the de Bruijn graph algorithm. Further description of the methodology was previously provided in detail^48^, as was a summary of the assembly statistics. Assemblies were generated for WMs, WFs, PKs and PQs (each biological replicate consisted of 5 individuals) to obtain appropriate RNA for RNA-seq.

### Read alignments and normalization of gene expression levels

SOAPaligner/soap2^70^ was used for short sequence alignment to read the sequences and map them to the reference sequences. The read coverage of one gene was used to calculate the level of expression of that gene. We obtained the expression levels of all the detected genes using this process. A read that was uniquely mapped to a gene was used to calculate the degree of expression. The level of gene expression was measured from the number of reads per kilobase of the exon region per million mapped reads (RPKM). The R package (http://www.r-project.org/) was used for statistical data expression and visualization.

### Differentially expressed genes (DEGs) and functional enrichment analyses

After calculating the rate of the expression of each gene, differential expression analysis was performed using edgeR^71^. After multiple tests, the false discovery rate (FDR) was used to determine the threshold for the P-value, and an FDR threshold of 0.01 and an absolute value of log2Ratio ≥ 1 were used to measure the significance of differences in gene expression in the sample. Using a method similar to that described by Zhang^72^, the differentially expressed genes were subjected to the analysis of GO and KEGG enrichment. Operational annotations for unigenes can be extracted from the analysis of Nr annotations. The GO annotation profile was checked with Blast2GO software^73^. The functional classification of unigenes was performed using WEGO software^74^. For DEGs, a Q-value <0.05 for GO terms and KEGG pathways were considered to indicate significant enrichment.

### Quantitative real-time PCR (RT-qPCR)

We designed primer pairs for each gene associated with the insulin signalling pathway with Primer3 v1.1.4 (Supplementary Table 2). Total RNA was extracted from the entire body of individuals of each caste (PQs, PKs, and WFs and WMs workers) using the RNAsimple Total RNA Kit (Tiangen). After the extraction of RNA, its quality and quantity (purity of protein and salt) were checked with a NanoReady spectrophotometer (Model: F-1100 made in China). To build the cDNA (the cDNA was held at -20 °C for further experiments) library, the FastKing RT Kit (Tiangen) was used. RT-qPCR was used to amplify the cDNA by using a CFX 96 instrument (Bio-Rad) with SuperReal PreMix Plus (Tiangen). All procedures were carried out in compliance with the manufacturer’s protocol. Since beta-actin (*RsACT*)^75,76^ was evaluated as the most reliable reference gene for *Reticulitermes* termites, it was selected as the reference^77^. The standard 2^−∆∆Ct^ method was used to calculate relative gene expression^78^. We conducted three biological and three experimental replicates (each replicate consisted of 5 individuals) for all RT-qPCR experiments.

### Statistical analysis

For the statistical analysis of the RT-qPCR experiments, SPSS Statistics 17.0 was used. Significance between groups was measured through a one-way variance analysis (ANOVA) followed by the Duncan post hoc test. All data in graphs are shown as the mean ± standard mean error, and all estimated P values <0.05 are given.

## Supplementary information

Supplementary information.

Supplementary Table S3.

## Data Availability

The datasets generated and analyzed during the current study are deposited under BioProject accession number PRJNA592596 at the NCBI. Any reasonable requests will be answered by the corresponding author.
