# Author Correction: Transcriptomic evidence that insulin signalling pathway regulates the ageing of subterranean termite castes

**DOI:** 10.1038/s41598-021-85420-1

**Published:** 2021-03-11

**Authors:** Haroon, Xiao-Ming Ma, Yu-Xin Li, Hong-Xin Zhang, Qing Liu, Xiao-Hong Su, Lian-Xi Xing

**Affiliations:** 1 Shaanxi Key Laboratory for Animal Conservation (Northwest University), Xi’an, 710069 People’s Republic of China; 2 Key Laboratory of Resource Biology and Biotechnology in Western China (Northwest University), Ministry of Education, Xi’an, 710069 People’s Republic of China; 3 College of Life Sciences, Northwest University, No. 229, North Taibai Rd., Xi’an, 710069 Shaanxi Province People’s Republic of China

Correction to: *Scientific Reports* 10.1038/s41598-020-64890-9, published online 18 May 2020

The original version of this Article contained errors.

The Article omitted a list of insulin-related genes identified in this study as differentially expressed. This list is now included in the Supplementary Table 3.

This change does not affect the conclusions of the Article.

