# Supplementary material for: Transcriptomic evidence that insulin signalling pathway regulates the ageing of subterranean termite castes
Source: Sci Rep. 2020 May 18;10:8187. doi: 10.1038/s41598-020-64890-9 (PMC7235038; doi:10.1038/s41598-020-64890-9)
Supplement: Supplementary file 1 — Supplementary information. [file 41598_2020_64890_MOESM1_ESM.docx]

Transcriptomic evidence that insulin signalling pathway regulates the ageing of subterranean termite castes

**Haroon**^3^**, Xiao-Ming Ma**^3^**, Yu-Xin Li**^3^**, Hong-Xin Zhang**^3^**, Qing Liu**^3^, **Xiao-Hong Su**^1 2 3^**, Lian-Xi Xing**^1 2 3^

^1^ Shaanxi Key Laboratory for Animal Conservation (Northwest University), Xi’an 710069, China.

^2^ Key Laboratory of Resource Biology and Biotechnology in Western China (Northwest University), Ministry of Education, Xi’an 710069, China.

^3^ College of Life Sciences, Northwest University, No. 229, North Taibai Rd., Xi’an, Shaanxi Province, 710069, P. R. China


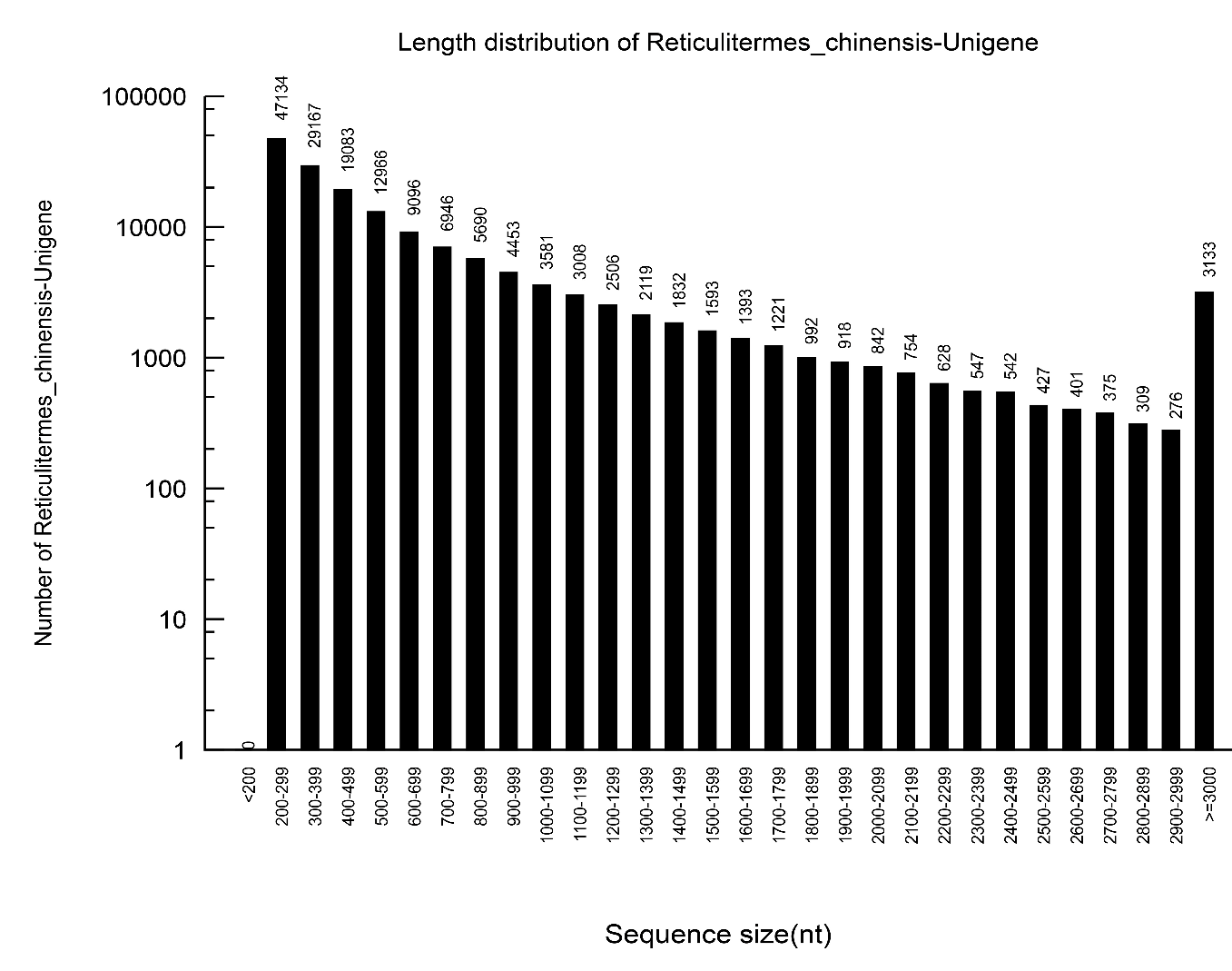


Supplementary Figure 1. Distribution of the lengths of the *R. chinensis* unigenes. The histogram presents the sequence length distribution for the identified significant matches. The x-axis indicates the sequence sizes from 200 nt to >3000 nt. The y-axis indicates the number of unigenes for every given size. The results of the sequence-length matches (with a cut-off E-value of 1.0E-5) in the Nr databases were greater among the longer assembled sequences.

Supplementary Table 1. Functional annotation of the *R. chinensis* transcriptome. BLASTX was used to query various protein databases and annotate 161,933 unigene sequences. All of the unigenes sequence against the Nr, Swiss-Prot, KOG, and KEGG databases.

| Total Unigenes | Nr | Swissprot | | KEGG | KOG | Annotation genes | | Without annotation gene |
| --- | --- | --- | --- | --- | --- | --- | --- | --- |
| 161,933 | 60,736 | 31,699 | 27,181 | | 28,003 | | 61,090 | 100,843 |


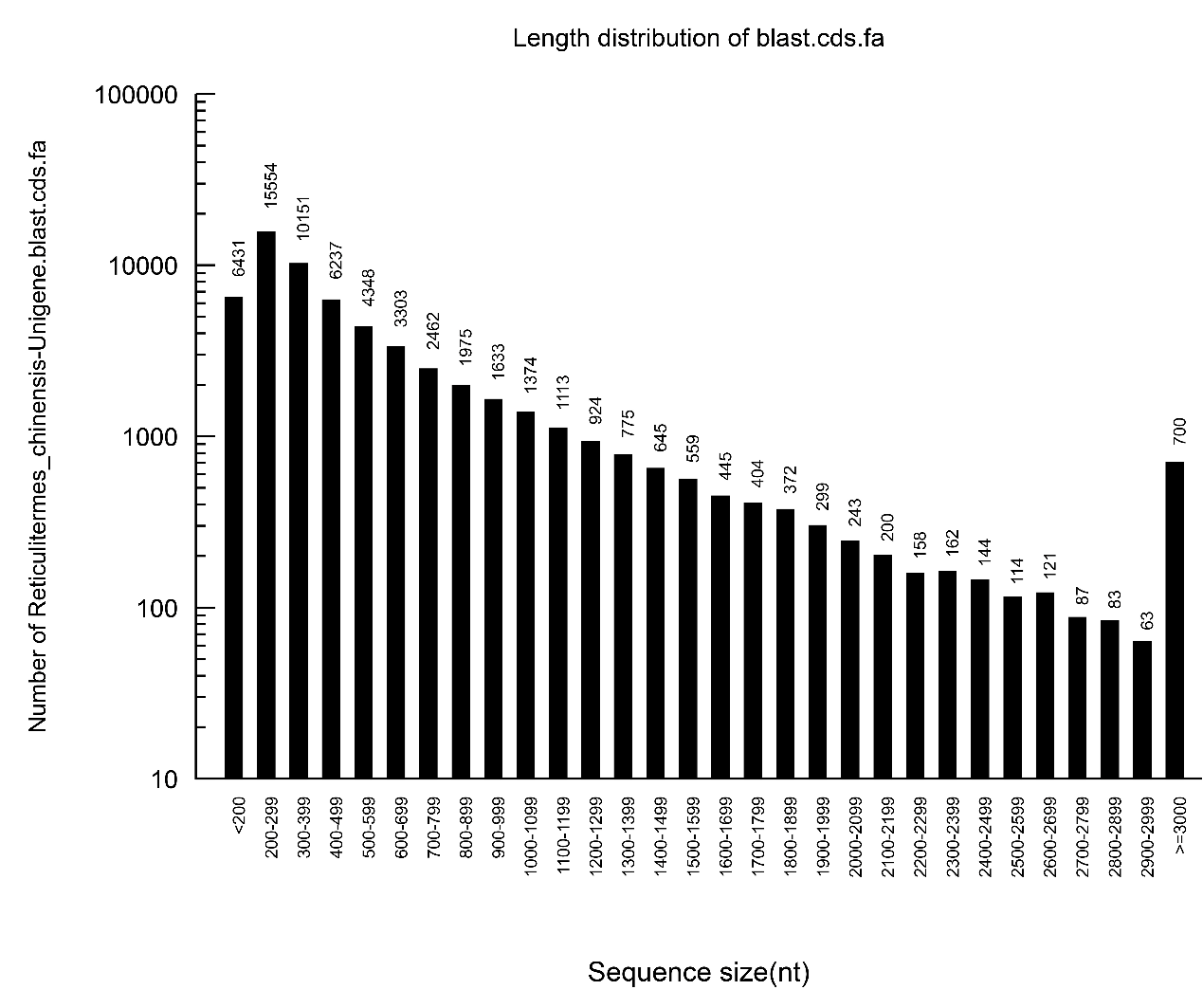


Supplementary Figure 2. Length distribution of Protein-Coding Region prediction (CDS) from BLAST. A total of 61,079 predicted unigenes using BLASTX histogram indicate the sequence-length distribution for significant matches that were found. The x-axis indicates the sequence size from 0 nt to >3000 nt. The y-axis indicates the number of unigenes for every given size.


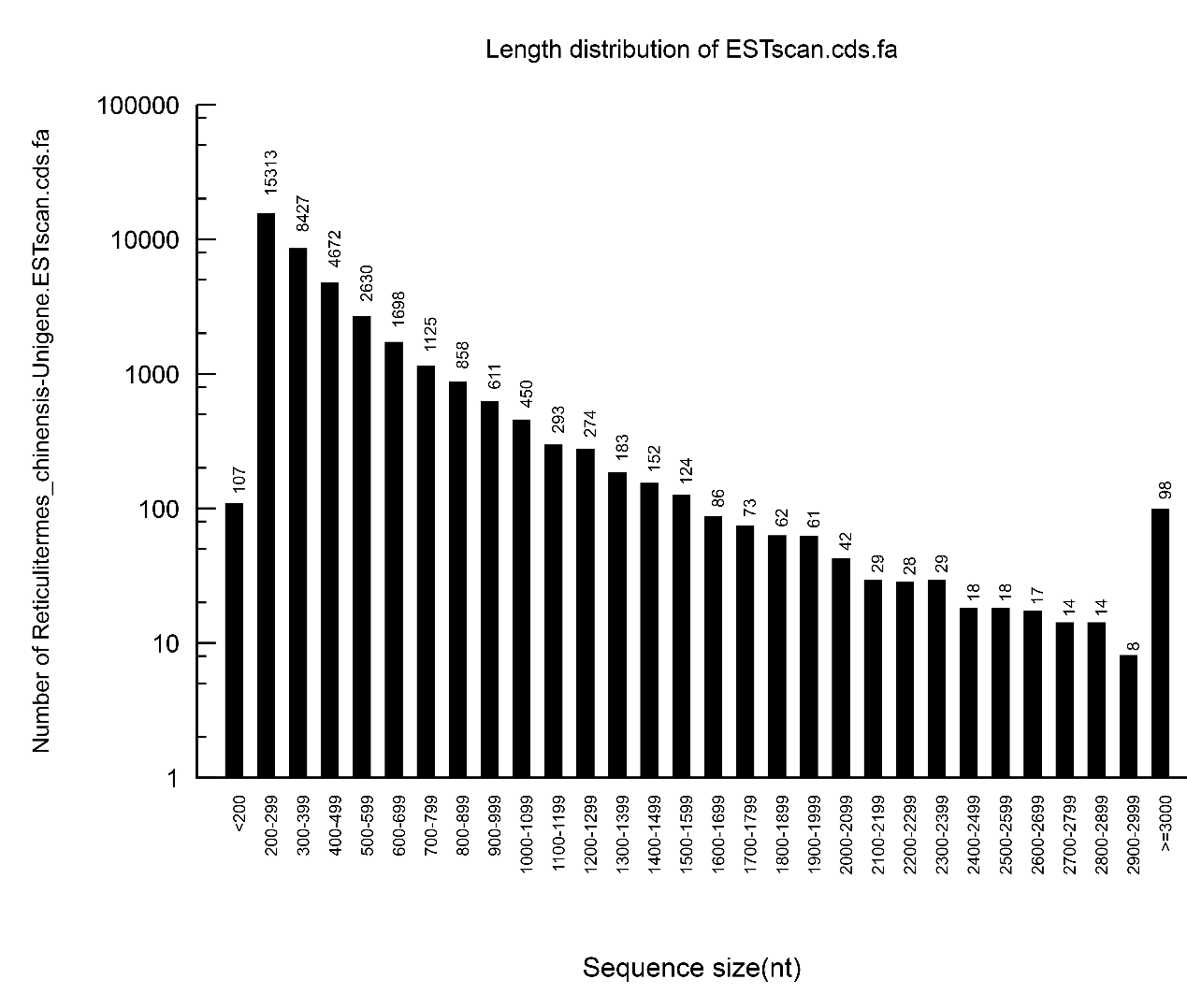


**Supplementary Figure 3. Length distribution of Protein-Coding Region prediction (CDS) from ESTcan.** A total of 37,514 predicted unigenes using ESTScan histogram indicate the sequence-length distribution for significant matches that were found. The x-axis indicates the sequence size from 0 nt to >3000 nt. The y-axis indicates the number of unigenes for every given size.

**Supplementary Table 2. The six selected genes and their primers used in RT-qPCR analyses.**

| Gene ID | Symbol | Primers sequences |
| --- | --- | --- |
|  | Beta-actin | Forward:CCCAACACAGCGTCTTACAA  Reverse:CAGATGTCCTCAGCTTCACG |
| Unigene 0082575 | *PdK1* | Forward:TCCTCCTCCTGCTACTGCTGAAG  Reverse:CGACATATGACGGAGTAGGTGGTG |
| Unigene 0034890 | *akt2-a* | Forward:CCAAGAAGTATGTCGAAAGAAGTCA  Reverse:TCTGTGAAACCATCTCCCAATTAAG |
| Unigene 0092210 | *Tsc2* | Forward:AGTGGTGCTAACATGCCTGC  Reverse:ACCTTCCAGCTGCTCTGACA |
| Unigene 0063105 | *mTOR* | Forward:GGCTTGAAGGGTGTTCCACA  Reverse:GCTTACCTGTAGGCGGCAAT |
| Unigene 0011832 | *EIF4E* | Forward:GGATCTCGTCTTGGCCGTCATTG  Reverse:AGCAACCTCGTGAGCCACTCC |
| Unigene 0155613 | *RPS6* | Forward:TCTATGACAAGCGCCTTGGAGAAG  Reverse:CTTGGACGGAGGCAGGTGGATC |

| 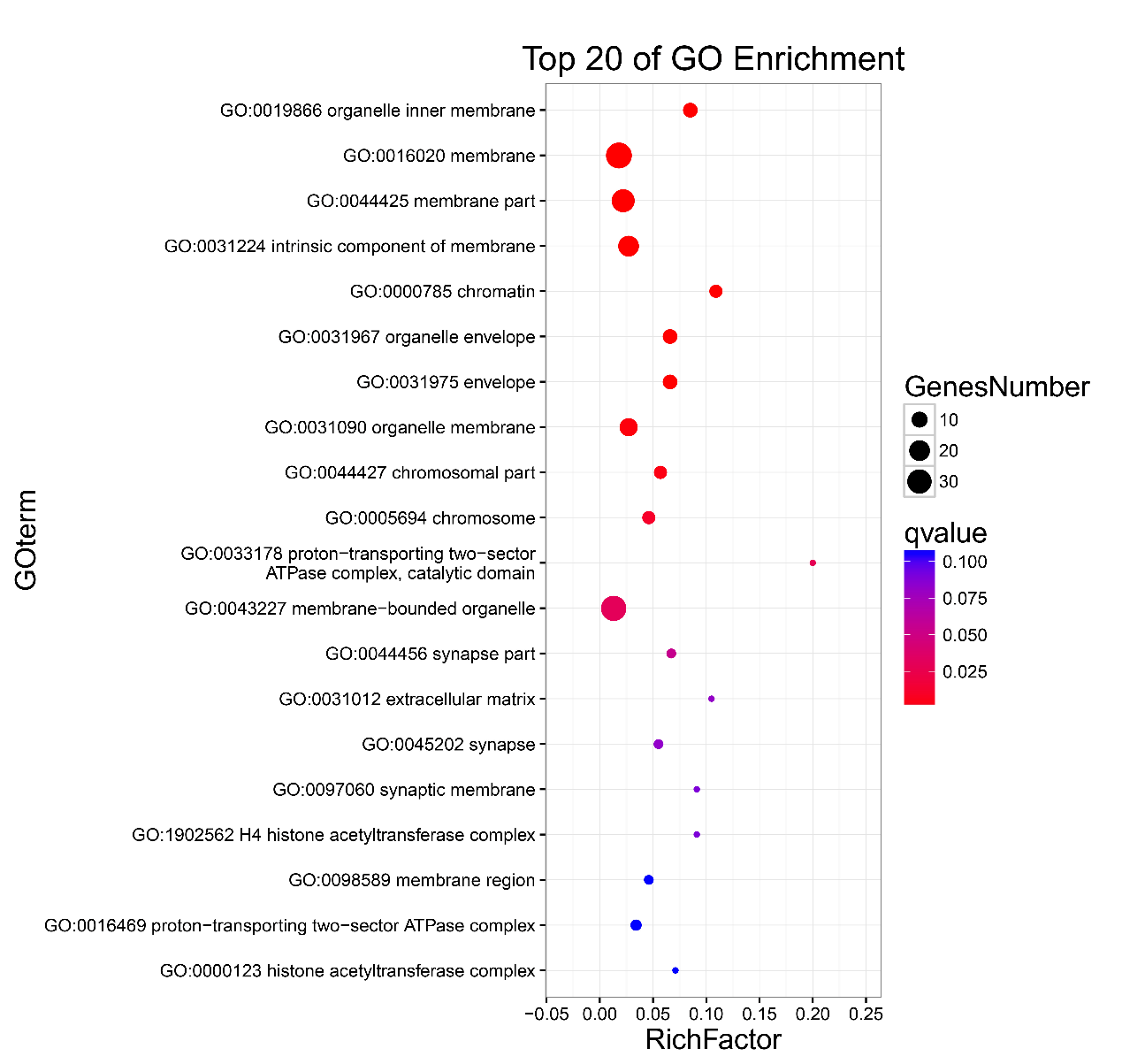  A |
| --- |
| 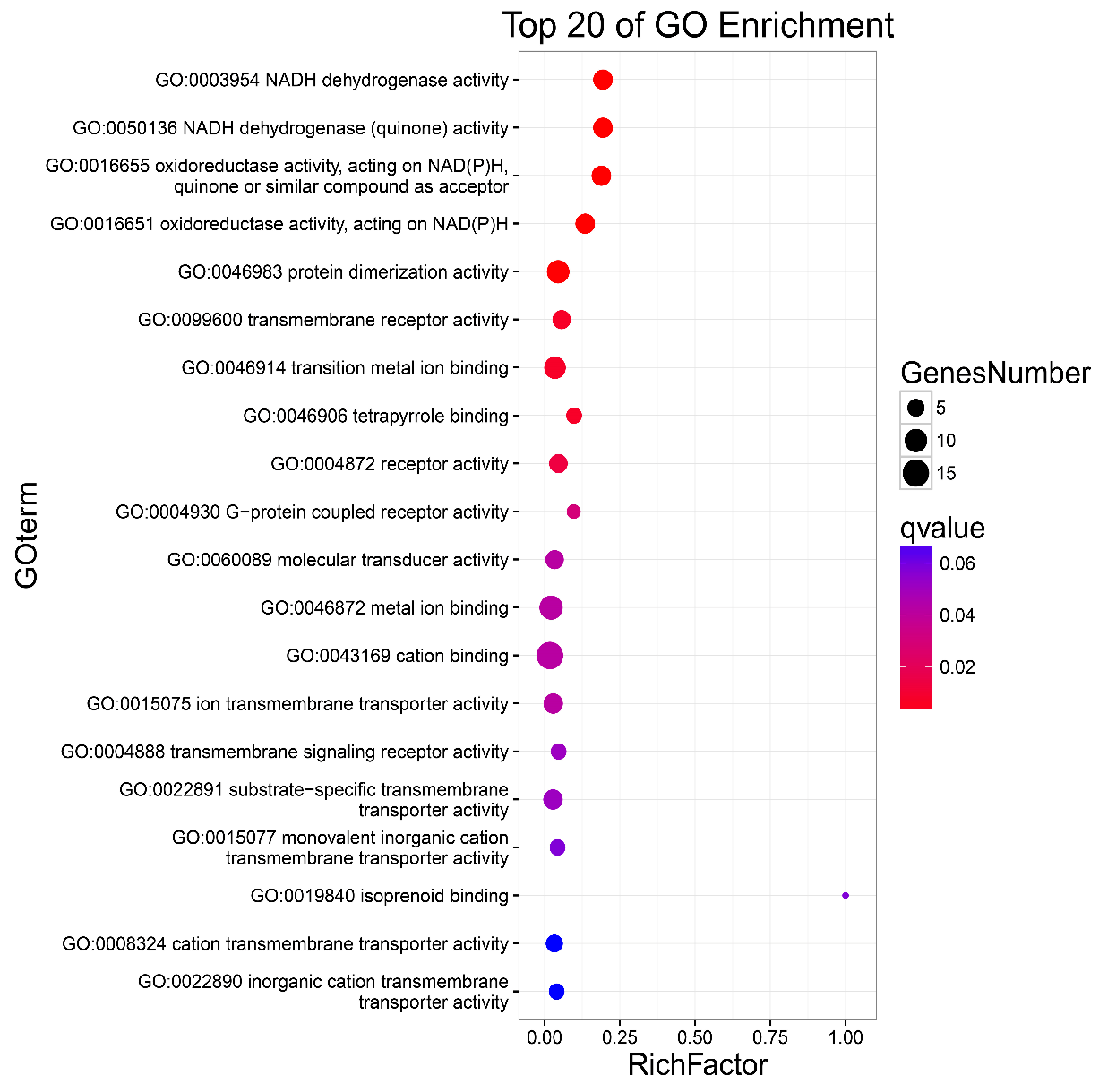  B |
| 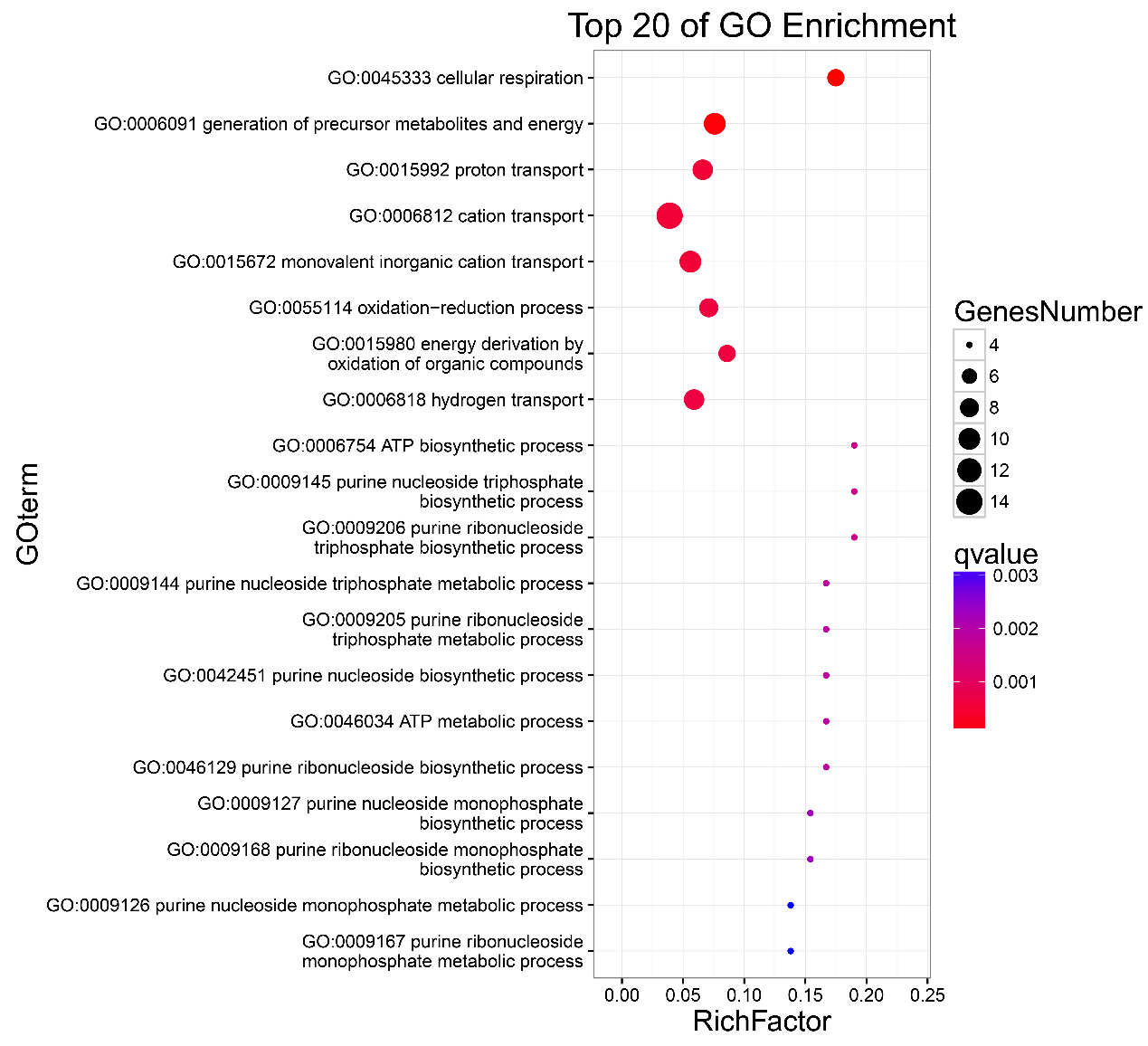  C |
| 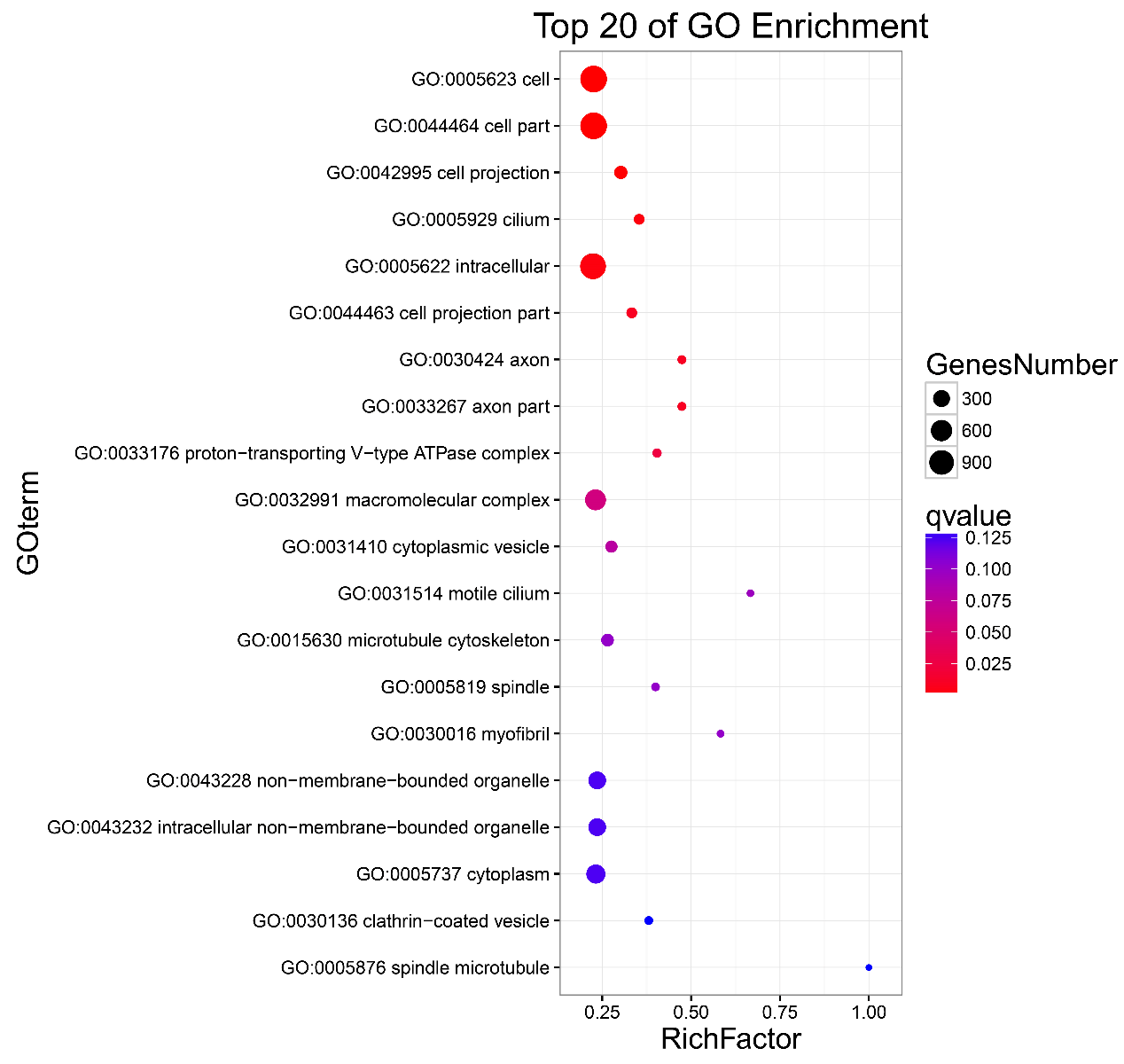  D |
| 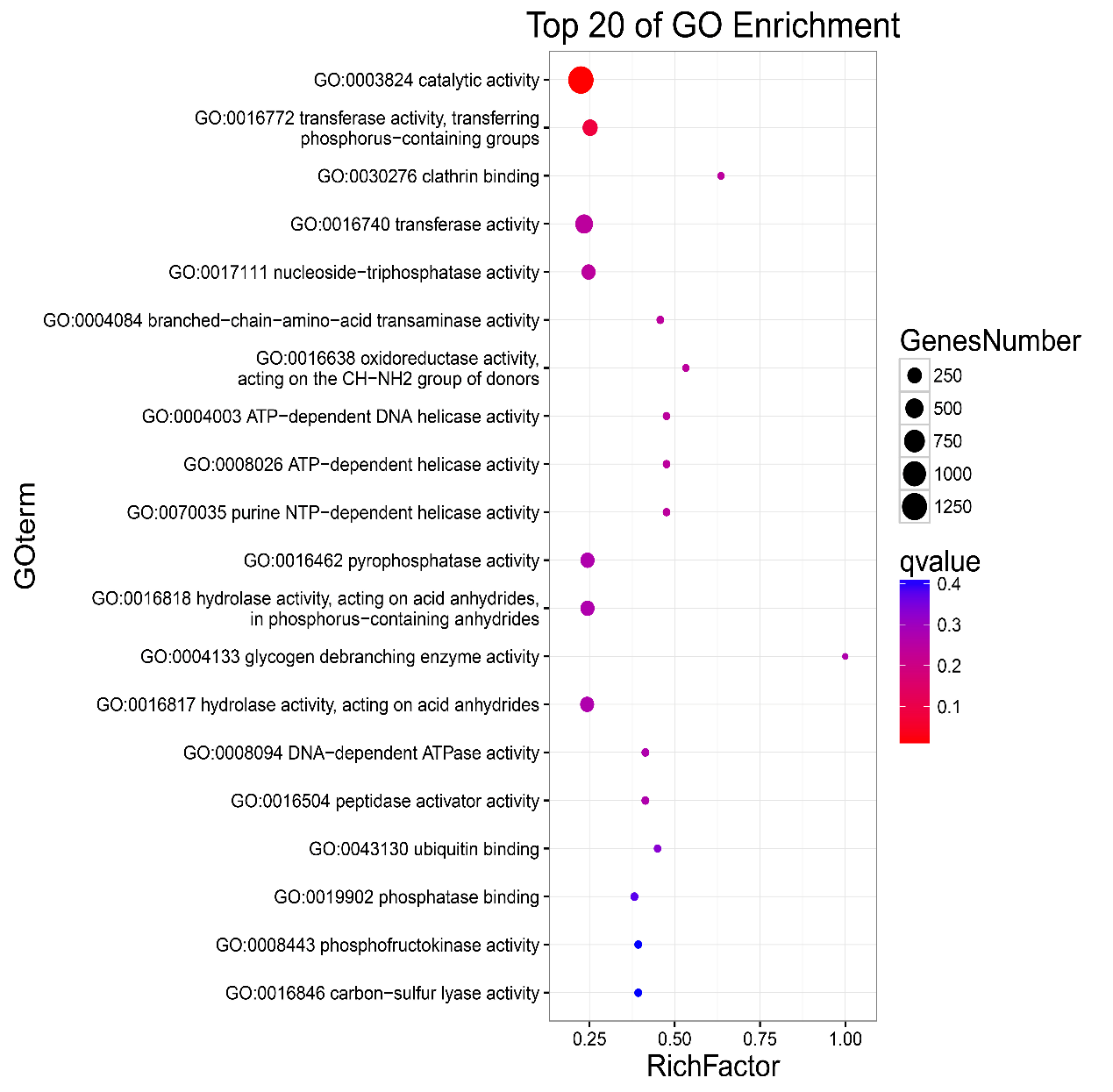  E |
| 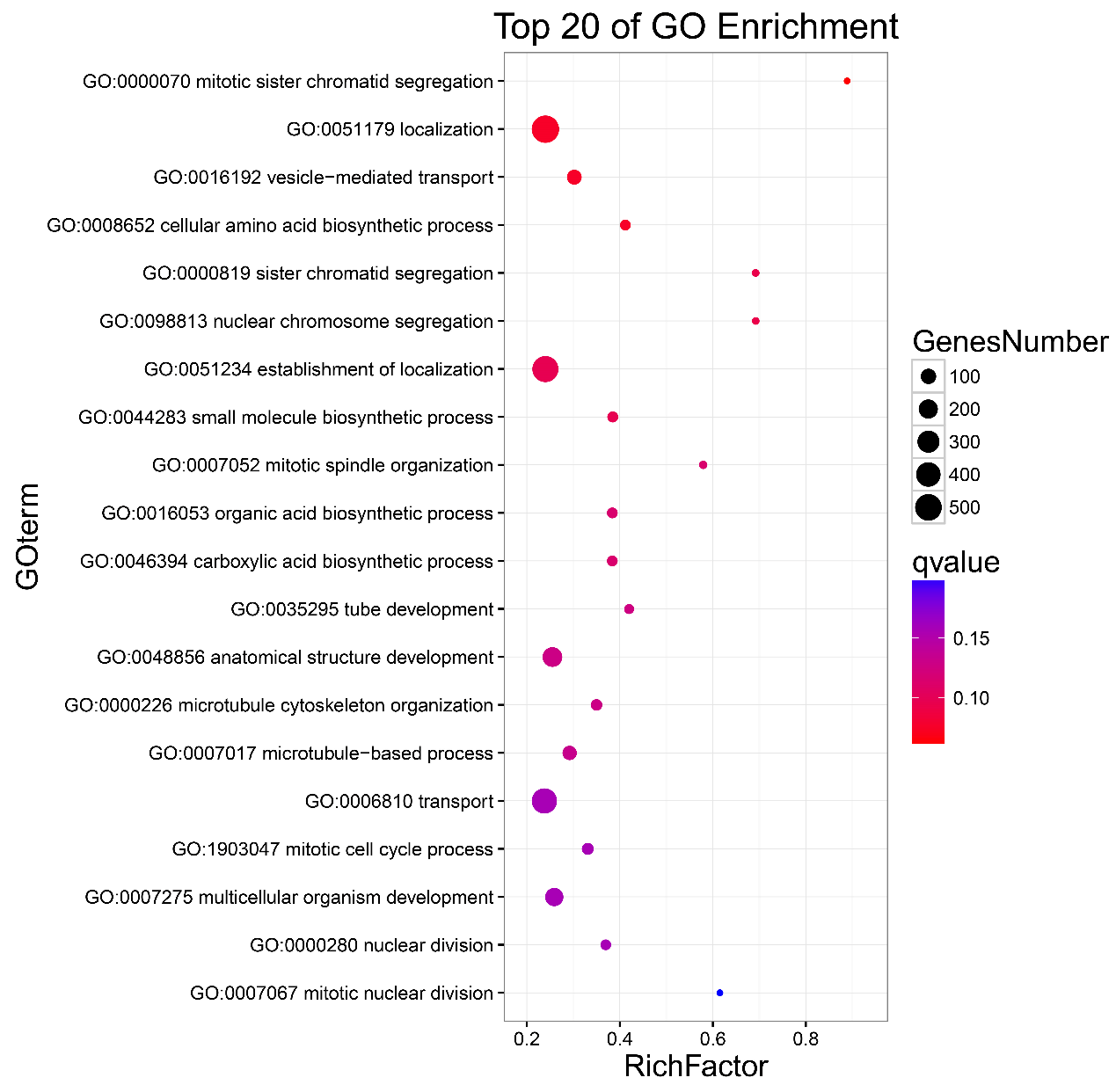  F |
| 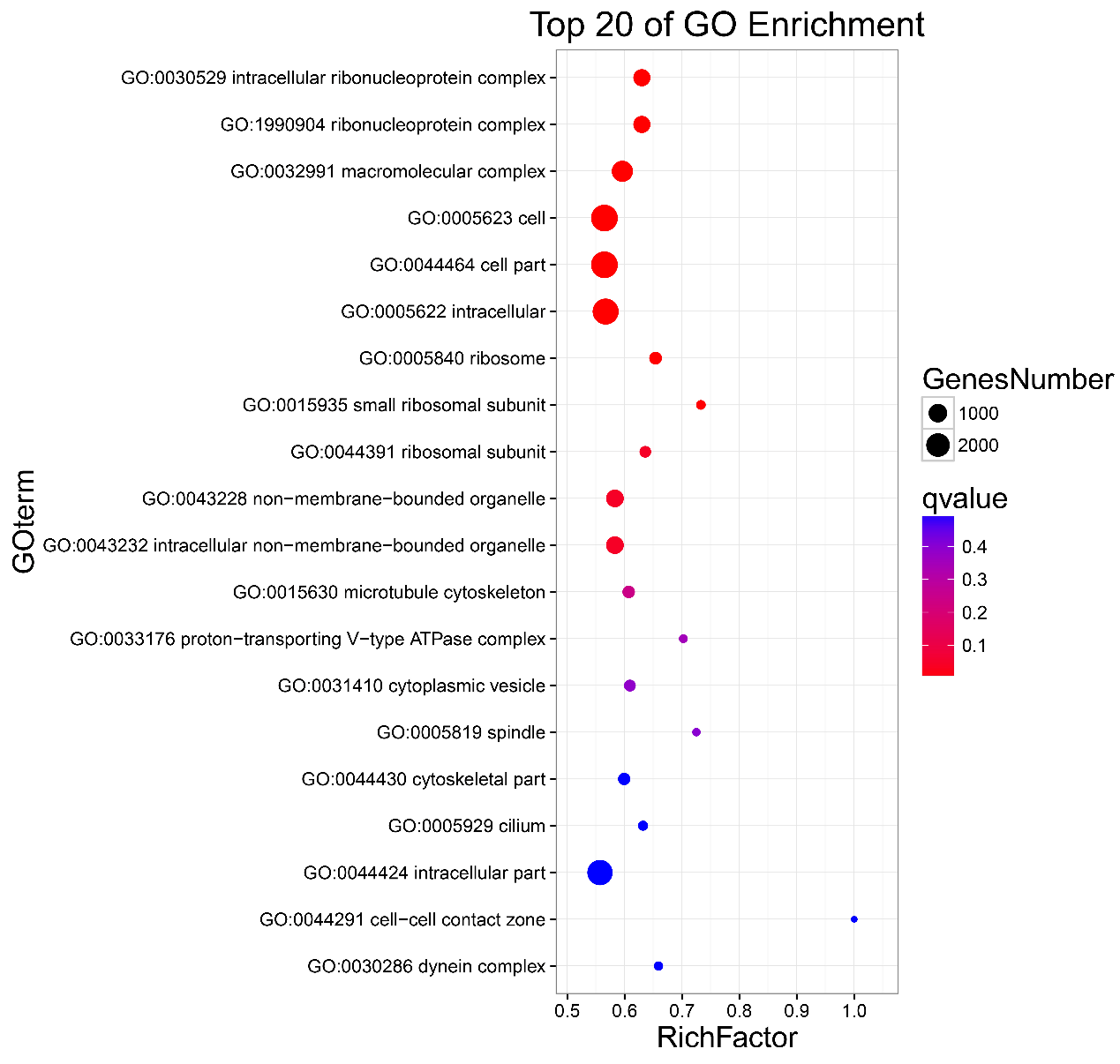  G |
| 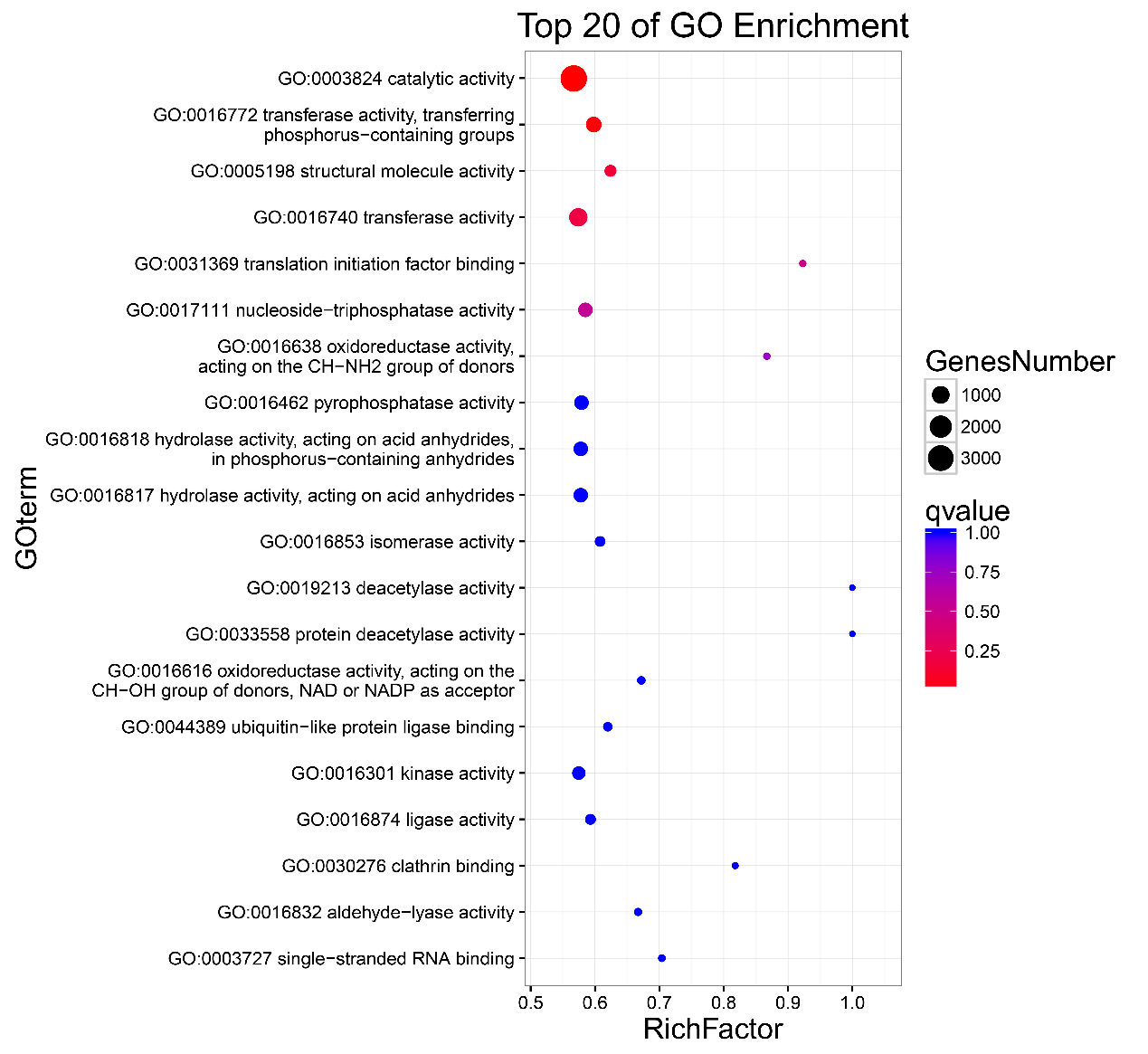  H |
| 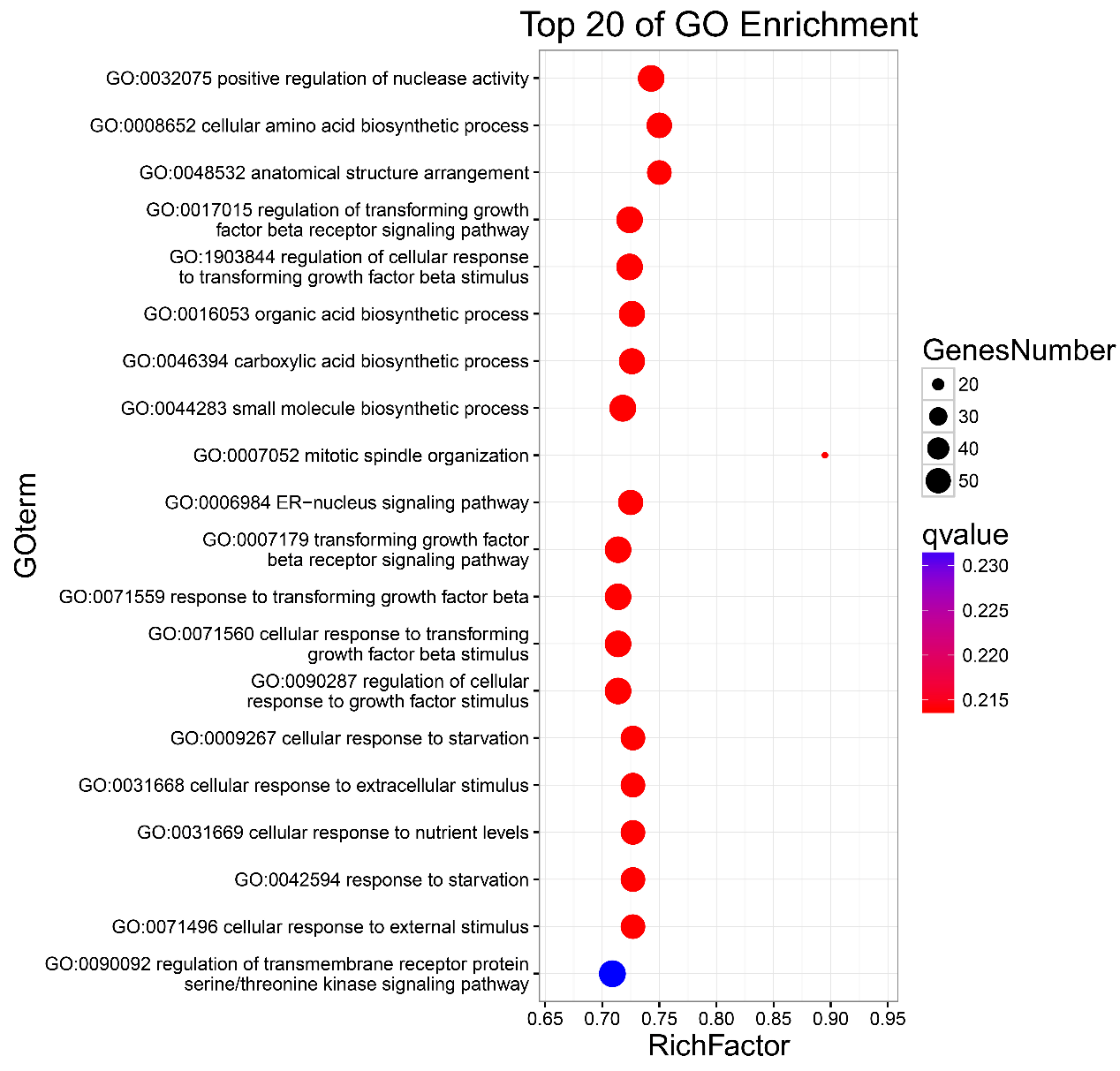  I |
| 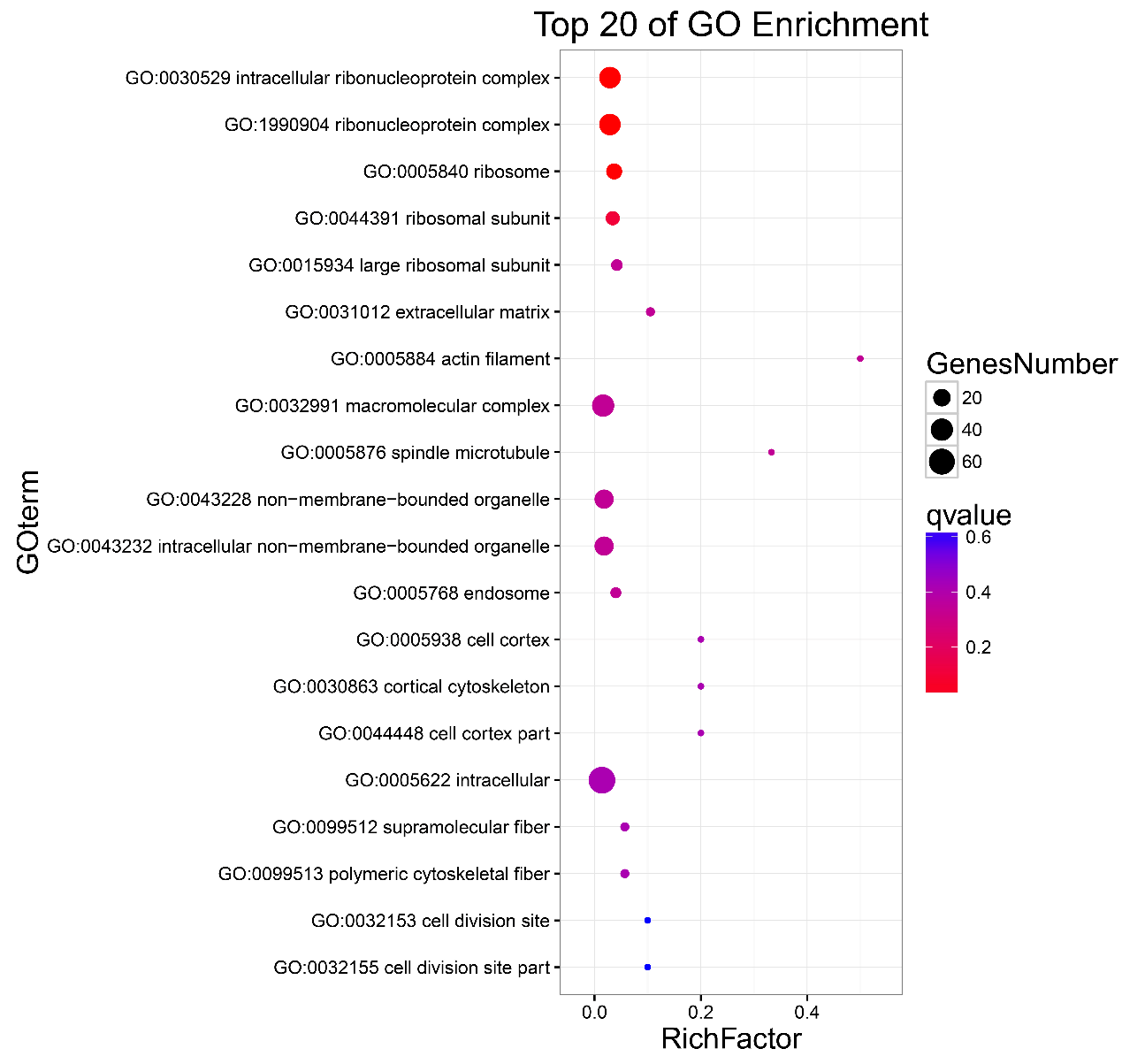  J |
| 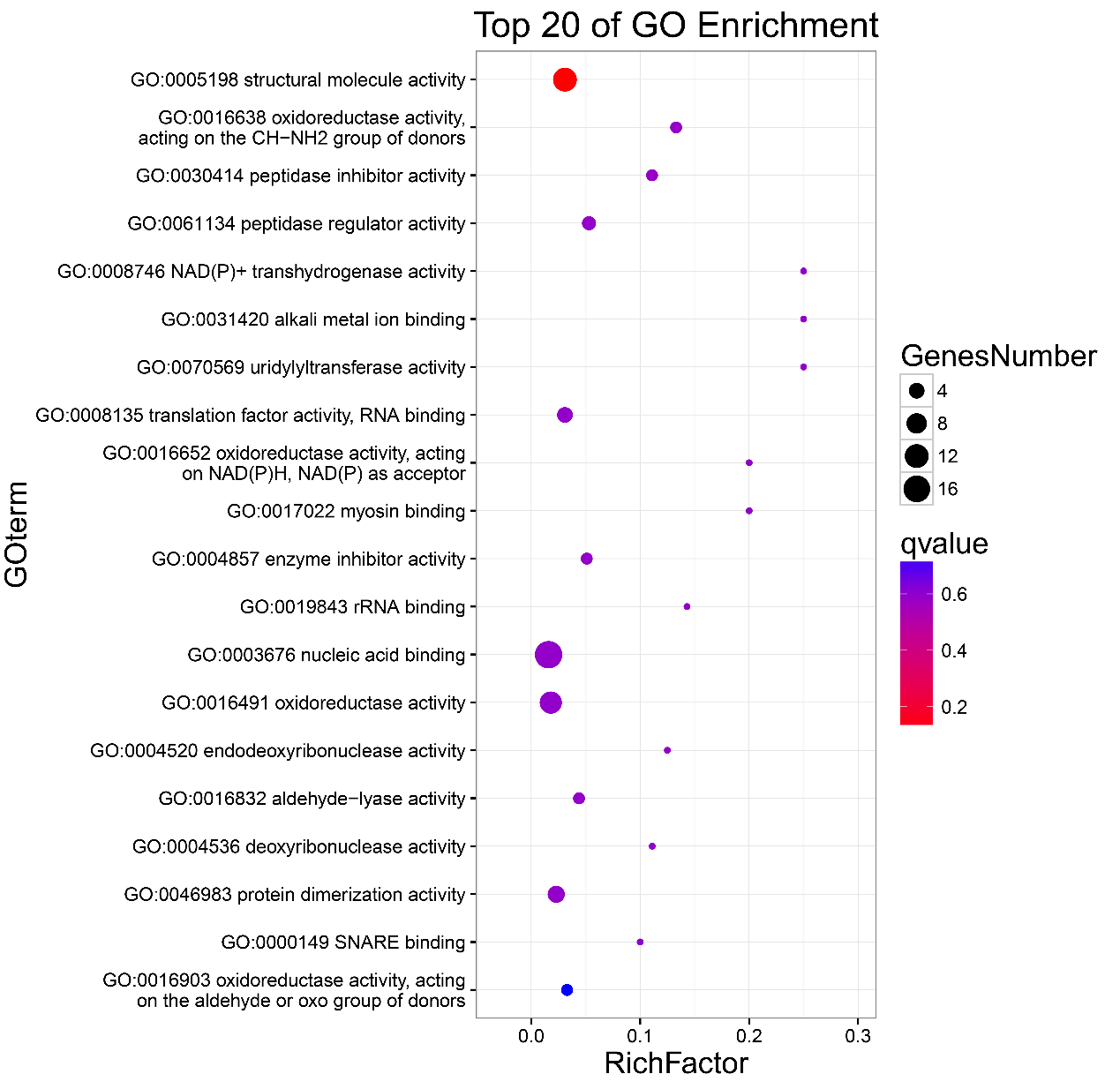  K |
| 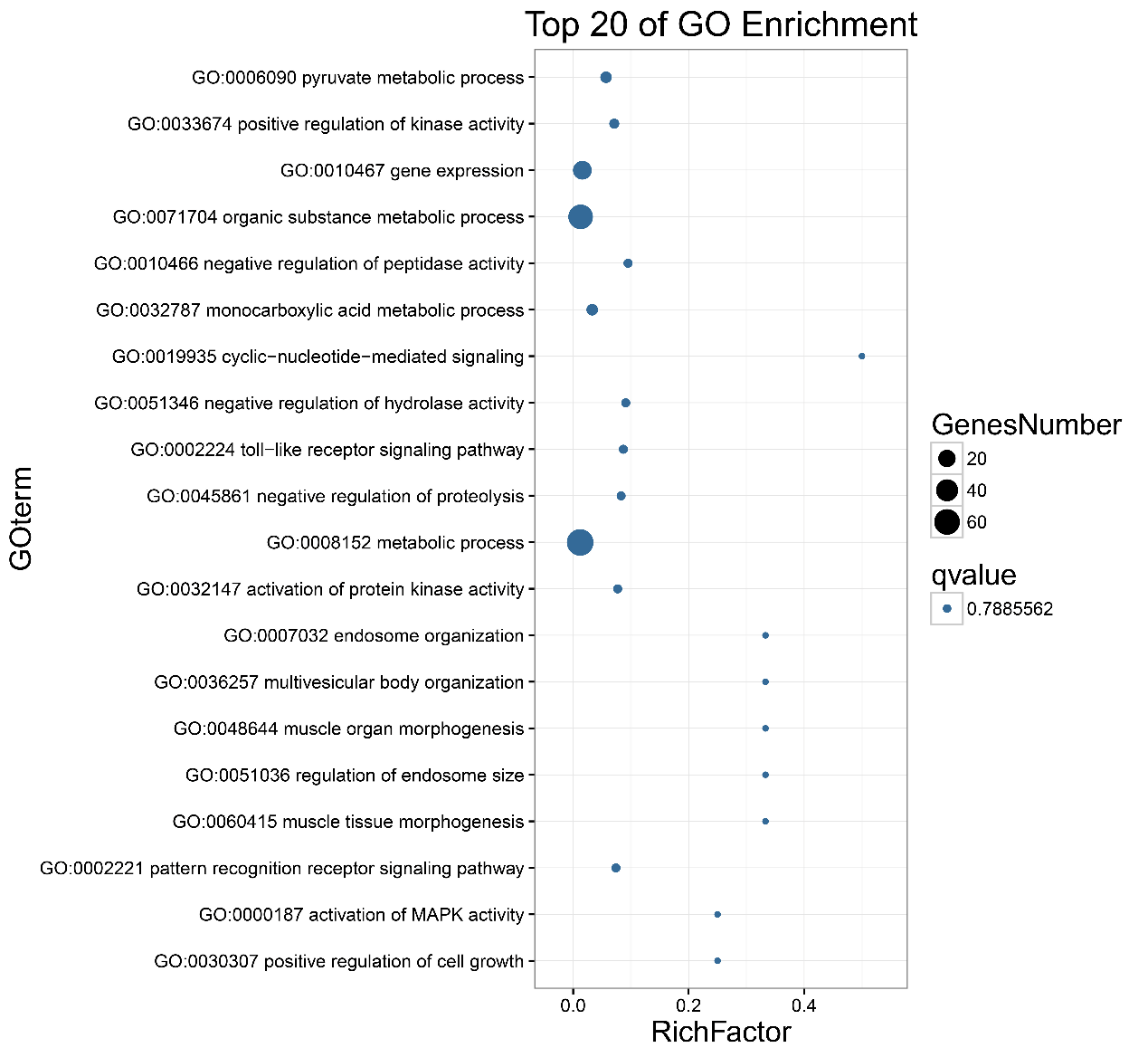  L |

**Supplementary Figure 4. Gene Ontology (GO) enrichment classification in each caste.** PK-VS-PQ GO enrichment (A, cellular components; B, molecular functions; C, biological process); PK-VS-WM GO enrichment (D, cellular components; E, molecular functions; F, biological process); PQ-VS-WF GO enrichment (G, cellular components; H, molecular functions; I, biological process) and WM-VS-WF GO enrichment (J, cellular components; K, molecular functions; L, biological process).
